# Hypothermia combined with extracellular vesicles from clonally expanded immortalized mesenchymal stromal cells improves neurodevelopmental impairment in neonatal hypoxic-ischemic brain injury

**DOI:** 10.1186/s12974-023-02961-0

**Published:** 2023-11-27

**Authors:** Nicole Labusek, Parnian Ghari, Yanis Mouloud, Christian Köster, Eva Diesterbeck, Martin Hadamitzky, Ursula Felderhoff-Müser, Ivo Bendix, Bernd Giebel, Josephine Herz

**Affiliations:** 1 Department of Pediatrics I, Neonatology and Experimental Perinatal Neurosciences, Center for Translational Neuro- and Behavioral Sciences (C-TNBS), University Hospital Essen, University Duisburg-Essen, Essen, Germany; 2 Institute for Transfusion Medicine, University Hospital Essen, University of Duisburg-Essen, Essen, Germany; 3 Institute for Medical Psychology and Behavioral Immunobiology, Center for Translational Neuro- and Behavioral Sciences (C-TNBS), University Hospital Essen, University of Duisburg-Essen, Essen, Germany

**Keywords:** Neonatal hypoxia–ischemia, Neonatal encephalopathy, Hypothermia, Mesenchymal stem/stromal cells, Extracellular vesicles, Long-term neurodevelopmental deficits, Neuroinflammation, Neuroregeneration

## Abstract

**Background:**

Neonatal encephalopathy following hypoxia–ischemia (HI) is a leading cause of childhood death and morbidity. Hypothermia (HT), the only available but obligatory therapy is limited due to a short therapeutic window and limited efficacy. An adjuvant therapy overcoming limitations of HT is still missing. Mesenchymal stromal cell (MSC)-derived extracellular vesicles (EVs) have shown promising therapeutic effects in various brain injury models. Challenges associated with MSCs’ heterogeneity and senescence can be mitigated by the use of EVs from clonally expanded immortalized MSCs (ciMSCs). In the present study, we hypothesized that intranasal ciMSC-EV delivery overcomes limitations of HT.

**Methods:**

Nine-day-old C57BL/6 mice were exposed to HI by occlusion of the right common carotid artery followed by 1 h hypoxia (10% oxygen). HT was initiated immediately after insult for 4 h. Control animals were kept at physiological body core temperatures. ciMSC-EVs or vehicle were administered intranasally 1, 3 and 5 days post HI/HT. Neuronal cell loss, inflammatory and regenerative responses were assessed via immunohistochemistry, western blot and real-time PCR 7 days after insult. Long-term neurodevelopmental outcome was evaluated by analyses of cognitive function, activity and anxiety-related behavior 5 weeks after HI/HT.

**Results:**

In contrast to HT monotherapy, the additional intranasal therapy with ciMSC-EVs prevented HI-induced cognitive deficits, hyperactivity and alterations of anxiety-related behavior at adolescence. This was preceded by reduction of striatal neuronal loss, decreased endothelial, microglia and astrocyte activation; reduced expression of pro-inflammatory and increased expression of anti-inflammatory cytokines. Furthermore, the combination of HT with intranasal ciMSC-EV delivery promoted regenerative and neurodevelopmental processes, including endothelial proliferation, neurotrophic growth factor expression and oligodendrocyte maturation, which were not altered by HT monotherapy.

**Conclusion:**

Intranasal delivery of ciMSC-EVs represents a novel adjunct therapy, overcoming limitations of acute HT thereby offering new possibilities for improving long-term outcomes in neonates with HI-induced brain injury.

**Supplementary Information:**

The online version contains supplementary material available at 10.1186/s12974-023-02961-0.

## Background

Neonatal encephalopathy (NE), caused by hypoxia–ischemia (HI) occurring around birth, is a devastating disease accounting for 23% of neonatal deaths worldwide [1] and substantial neurodevelopmental impairment [2]. The introduction of therapeutic hypothermia (HT) as standard clinical care two decades ago was a game changer with regard to mortality and severe motor disability. However, approximately 30% of survivors still suffer from long-term neurological sequela such as sensory, cognitive and neuropsychological impairment, becoming evident specifically at school age and beyond [2–9].

Limited efficacy of HT is mainly attributed to its short therapeutic window of 6 h, i.e., suppressing excitotoxicity, oxidative stress and acute cell death [10, 11]. However, effects on secondary and tertiary disease phases, characterized by ongoing inflammation, gliosis, impaired oligodendrocyte maturation and myelination overlapping with disturbed endogenous regeneration (e.g., angiogenesis) are limited. For instance, treatment with HT started at 3 h after global cerebral ischemia only partially suppressed microglial activation in fetal sheep [12]. In rodents it was further shown that HT provides only selective protection to particular brain structures. While neuronal loss is reduced in hippocampal CA1–CA3 regions, protection of striatal neurons is limited [13–15]. Furthermore, clinical and pre-clinical studies suggested limited effects on white matter injury and oligodendrocyte maturation [16–21]. White matter development is key for long-term neurodevelopment, as demonstrated by correlations between alterations in the white matter microstructure and neurodevelopmental outcome in children with NE [17, 20, 21]. This is supported by previous work in a pre-clinical HI model, revealing protection from motor but not cognitive deficits, associated with reduced myelination [15, 18].

To improve efficacy of HT, various pharmacological agents have been evaluated in a variety of pre-clinical trials [10, 11]. However, none of these agents could be transferred into clinical practice due to lack of efficacy in first clinical trials [22–24]. According to the multimodal mechanisms of action, mesenchymal stromal cell (MSC)-based therapies have been suggested as a promising alternative therapy [10, 11]. However, the cells’ plasticity, heterogeneity and their limited life span present major hurdles for clinical translation. Extracellular vesicles (EVs) derived from MSCs can overcome limitations of cell therapies, since the risk of changing their function depending on their microenvironment can be neglected. The neuroprotective potential of MSC-EVs has been proven in several perinatal brain injury models [25–32]. The challenge of MSC-EV heterogeneity [33, 34] can be mitigated by using EVs from clonally expanded and immortalized MSCs (ciMSCs). Recent work in a rodent model of neonatal HI showed that intranasal delivery of ciMSC-EVs provides similar neuroprotection as EVs from primary MSCs [31].

In the present study, we hypothesized that intranasal application of ciMSC-EVs overcomes the above-mentioned current limitations of HT. Using a translational rodent model of neonatal HI and HT, subacute effects on neuronal degeneration, neuroinflammation, oligodendrocyte maturation and neuroregeneration were analyzed. Long-term neurodevelopmental outcome in terms of cognitive function, anxiety-related behavior and hyperactivity were characterized by behavioral testing.

## Methods

### ciMSC growth, ciMSC-EV preparation and characterization

Cells of the primary human MSC stock were initially raised from a sample of a bone marrow graft of a healthy donor in an anonymized manner after informed consent according to the Declaration of Helsinki. This MSC stock (41.5) was used for immortalization followed by clonal expansion. In the present work, we applied the same EV preparation of the same clonal ciMSC-line as in our recent study [31]. Briefly, EVs were prepared from ciMSC conditioned media (Dulbecco’s modified Eagle medium supplemented with 10% human platelet lysate), harvested every 48 h between 34 and 37 passages [31]. EVs were prepared from thawed pooled conditioned media according to an established standard procedure, i.e., by polyethylene glycol 6000 precipitation followed by ultracentrifugation as described in detail before [35–37]. Obtained EV samples were characterized in accordance with the recommendation of *Minimal Information for Studies of Extracellular Vesicles 2018* criteria [38], including nanoparticle tracking analysis and imaging flow cytometry-based analyses of EV surface marker expression. Samples were further analyzed for their immunomodulatory capacity in a multi-donor mixed lymphocyte reaction assay, as described previously [33, 39, 40].

### Animal care and group allocation

Experiments were performed in accordance with the Animal Research Reporting of in Vivo Experiments (ARRIVE) guidelines with governmental approval by the State Agency for Nature, Environment and Consumer Protection North Rhine-Westphalia. C57BL/6J mice were bred in house and kept under a 12-h light/dark cycle with food and water ad libitum. Bodyweight of pups was recorded at postnatal day 9 (P9), P10, P11, P12 and P16 and weekly after weaning. A total of 104 C57BL/6 mice (*n* = 51 female and *n* = 53 male) derived from 14 litters were enrolled. For all analyses, animals per litter and experiment were randomly assigned to 4 experimental groups: sham *n* = 23 (11 female, 12 male), HI / normothermia (NT) / vehicle *n* = 28 (15 female, 13 male), HI / hypothermia (HT) / vehicle *n* = 25 (12 female, 13 male), HI / HT / ciMSC-EV *n* = 28 (13 female, 15 male) prior to intervention. To control for potential influence of weight and sex, a stratified randomization was performed followed by simple randomization within each block to assign pups to individual groups. Individuals involved in data analysis knew the animals’ designation but were blinded to group assignment. In total 11 animals exposed to HI (4 female, 7 male) died with random distribution between treatment groups (Additional file 1: Table S1). The mortality rate of 13.6% of all animals exposed to HI is within the range of our previous reports in the same experimental model [15, 18]. Details about group allocation and mortality rates are provided in Additional file 1: Table S1.

### Neonatal hypoxia–ischemia

Hypoxic-ischemic (HI) brain injury was induced as previously described [15, 18, 27, 31, 41, 42]. In brief, nine-day-old mice were exposed to HI through occlusion of the right common carotid artery through cauterization (high-temperature cauter, 1200 °C, Bovie, USA) under isoflurane anesthesia (1.5–4 Vol%), followed by 1 h hypoxia (10% O_2_) in an airtight oxygen chamber (OxyCycler, USA) after 1 h recovery with their dams (Additional file 1: Fig. S1A). To maintain nesting temperature, mice were placed on a warming mat during hypoxia according to our previously established HI model in neonatal mice [15, 18]. Sham animals received anesthesia and neck incision only. Perioperative analgesia was ensured by subcutaneous administration of 0.1 mg/kg buprenorphine.

### Therapeutic hypothermia and ciMSC-EV treatment

As previously described, HT was applied immediately following HI for 4 h at T_rectal_ = 32 °C [15, 18] (Additional file 1: Fig. S1A). Briefly, mice were placed on a custom-made thermo-plate with temperature control by water circulation to achieve a mean body core temperature of 32 °C [15]. Control animals (normothermia, NT) were kept on a warming mat to maintain physiological body core temperature of 35–36 °C [15]. ciMSC-EVs (1 × 10^5^ cell equivalents/g bodyweight) were administered intranasally in 2 × 2.5 µl/g body weight per nostril at 24 h, 72 h and 120 h post HI [31] (Additional file 1: Fig. S1A). Vehicle-treated control animals received the same volume of 0.9% NaCl at the same point in time. Thirty minutes prior to EV/vehicle treatment, 2.5 µl hyaluronidase (100U, Sigma Aldrich) were applied to each nostril as previously described [18, 31]. This pretreatment was performed to enhance EV uptake due to permeability-enhancing effects, facilitating diffusion and absorption of drugs [43, 44].

### Tissue preparation for assessment of neurodegeneration, neuroinflammation and neuroregeneration

Seven days after HI, mice were deeply anesthetized with chloral hydrate and transcardially perfused with ice-cold PBS. Brains were removed and snap frozen on dry ice. Tissues at the striatal level (+ 0.1 to + 0.5 mm from bregma) were used for analyses via immunohistochemistry, real-time PCR and western blot (Additional file 1: Fig. S1B).

### Immunohistochemistry

Analyses were performed on 20 µm cryostat tissue sections taken at the level of the striatum between + 0.1 mm and + 0.3 mm from bregma. For neuronal, oligodendrocyte and vessel densities, neuronal nuclei (NeuN), oligodendrocyte transcription factor 2 (Olig2) and cluster of differentiation 31 (CD31) were stained, respectively. Astrogliosis and microglia activation were evaluated by staining of glial fibrillary acidic protein (GFAP) and ionized calcium-binding adaptor protein-1 (Iba-1). Total leukocyte and neutrophil infiltration was analyzed following anti-CD45 and anti-Ly6G staining. For analyses of vascular injury and localization of neutrophils in relation to the vasculature, sections were co-stained for the basement membrane marker Laminin and Ly6G. Endothelial and oligodendrocyte proliferation were assessed in Ki67 immunostainings combined with CD31 or Olig2. Oligodendrocyte maturation was evaluated by co-staining of Olig2 with platelet-derived growth factor receptor alpha (PDGFR-alpha, immature oligodendrocytes) or adenomatous polyposis coli, clone CC1 positive (referred as CC1, mature oligodendrocytes). Detailed information on primary and secondary antibodies is provided in Additional file 1: Table S2.

Staining of tissue sections were performed according to previously published protocols [18, 27, 31, 41, 42]. Briefly, sections were thawed at 37 °C for 15 min followed by fixation in 4% paraformaldehyde (PFA) for NeuN, Olig2, Ki67 (host rat), PDGFR-alpha or in ice-cold acetone/methanol for CD31, Ki67 (host rabbit), CD45, Ly6G each for 5 min. For Iba-1/GFAP co-staining, sections were incubated with 4% PFA overnight at 4 °C, followed by antigen retrieval in sodium citrate buffer (10 mM tri-sodium citrate, 0.05% Tween-20; pH 6.0) at 100 °C for 30 min. Unspecific antibody binding was blocked by incubation with 1% bovine serum albumin, 0.3% cold fish skin gelatin (Sigma Aldrich, Germany), 0.2% Tween-20 in PBS for 1 h at room temperature followed by primary antibody incubation overnight at 4 °C. Antibody binding was visualized by incubation with appropriate anti-rat/mouse/rabbit Alexa Flour 488 and Alexa Flour 555 conjugated secondary antibodies (all:1:500, Thermo Scientific, Germany) for 1 h at room temperature. Nuclei were counterstained with 4′,6-diamidino-2-phenylindole (Dapi, 100 ng/ml; Molecular Probes, USA).

Confocal imaging with the 20 × objective (A1plus, Eclipse Ti, with NIS Elements AR software, Nikon, Germany) was used to generate z-stacks of 14 µm thickness (2 µm focal plane distance). Software-based quantification was performed in maximal intensity projection images of 3 ROIs in the striatum, 3 ROIs in the cortex and 4 ROIs in the white matter (Additional file 1: Fig. S1C). For NeuN, Olig2, CD31 and Ki67, unbiased software-based object counting was used. Single object counting was not possible for CD45, Iba-1 and GFAP staining due to intensive local accumulation of leukocytes, microglia activation and glial scar formation by astrocytes in severely affected animals. Therefore, positively stained areas were quantified as a measure of cell densities. Vascular injury, revealed by basement membrane disruption [41] was analyzed by quantification of positively stained areas for Laminin. Ly6G^+^ cells were counted manually, because automated software-based quantification tools are unable to identify single cells in regions of strong accumulation. For assessment of proliferating endothelial cells and oligodendrocytes, CD31/Ki67^+^ and Olig2/Ki67^+^ cells were counted. Maturation of oligodendrocytes was determined by quantification of Olig2/PDGFR-alpha and Olig2/CC1 double-positive cells.

### RNA and protein isolation

For RNA and protein isolation, 160-µm-thick tissue sections of the ipsilateral hemisphere were collected at the striatal level (0.3 mm to 0.5 mm from bregma). RNA and proteins were isolated via the NucleoSpin^®^ RNA/Protein Kit (Macherey–Nagel, Germany) according to the manufacturer’s instructions. Isolated RNA was eluted in 40 µl RNAse-free water and quantified using the NanoDrop Spectrophotometer (Peglab, Germany). Proteins were dissolved in 50 µl Protein Solving Buffer—Tris (2-carboxyethyl) phosphine hydrochloride (PSB-TCEP) provided by the Kit followed by denaturation at 95 °C. RNA and protein samples were stored at − 80 °C (RNA) and -20 °C (protein) until further processing.

### mRNA expression analysis

mRNA expression was analyzed according to our previous reports [27, 31]. 1.5 µg of total RNA and TaqMan reverse transcription reagents (Applied Biosystems/Thermo Fisher Scientific) were used to synthesize first strand complementary DNA. Real-time polymerase chain reaction (PCR) was performed in duplicates in 96 well-optical reaction plates for 40 cycles with each cycle at 94 °C for 15 s and 60 °C for 1 min using the StepOnePlus Real Time PCR system (Applied Biosystems/Thermo Fisher Scientific). PCR products were quantified using assay on demand primers and fluorogenic reporter oligonucleotide probes (Applied Biosystems/Thermo Fisher Scientific, Additional file 1: Table S3). Ct values were normalized to the housekeeping gene beta-2-microglobulin [Δct = ct (target gene) – ct (beta-2-microglobulin)] and related to the mean of sham animals using the ΔΔCT formula. Fold change values were calculated.

### Western blot analysis

Protein concentration was quantified by using the Protein Quantification Assay (Macherey–Nagel, Germany), followed by protein separation on 12.5% and gradient SDS polyacrylamide gels. Separated proteins were transferred to nitrocellulose membranes (0.2 µm, Amersham, USA) at 4 °C overnight. Equal loading of 7.5 µg / lane and transfer of proteins was confirmed by staining of membranes with Ponceau S solution (Sigma Aldrich). Nonspecific binding was blocked by incubation in 5% nonfat milk powder (Cell Signaling, USA), 0.1% Tween-20 in Tris-buffered saline (TBS) followed by incubation with the following primary antibodies: rabbit anti-NeuN, mouse anti-microtubule-associated protein 2 (MAP2), goat anti-vascular cell adhesion molecule-1 (VCAM-1), biotinylated goat anti-mouse intercellular adhesion molecule-1 (ICAM-1), rabbit anti-Iba-1, mouse anti-GFAP, mouse anti-myelin basic protein (MBP), and rabbit anti-glutaraldehyde-3-phosphate dehydrogenase (GAPDH, Additional file 1: Table S4) each in blocking solution at 4 °C overnight. Membranes were incubated with appropriate peroxidase-conjugated secondary antibodies (all 1:5000, DAKO, Denmark) in blocking solution at room temperature for 1 h followed by chemiluminescent detection with the enhanced chemiluminescence prime western blotting detection reagent (Amersham, GE Healthcare Life Science, USA). For ICAM-1, the Vectastain ABC-HRP Kit (Vector Laboratories, USA) was used according to the manufacturers’ instructions. For visualization and densitometric analysis, the ChemiDocXRS + imaging system and ImageLab software (Bio-Rad, Germany) were used.

### Long-term functional outcome

Behavioral testing was performed from postnatal day 44–52, i.e., day 35 to day 43 after HI according to previously described protocols [15, 18, 25, 45] (Additional file 1: Fig. S1D). Shortly, animals were transferred to an inverted light/dark cycle after weaning. Behavioral testing was carried out during the dark phase in a low noise environment (behavioral unit). Animals’ behavior was recorded by using an automatic tracking system (Noldus Ethovision XT15, Germany). All test devices were cleaned with 70% ethanol between each trial to minimize interference of odorants. Spontaneous exploration, activity and anxiety-related behaviors were assessed in the Elevated Plus Maze (EPM) and Open Field (OF) maze [46, 47]. EPM was performed on the first day of testing. Mice were placed in the center of the maze and behavior was recorded for a duration of 5 min. The mean velocity, the total distance moved, the time spent in the open arms, the proportion of distance moved in the open arms and the frequency of head dipping in the open arms was quantified. The OF test was performed on the second day. Mice were placed into the center of an open field arena and movement was tracked for 5 min. To evaluate behavior, mean velocities, total distances moved and the time the animals moved in the central area of the box was analyzed. To study learning capabilities and visual-spatial memory a modified Barnes Maze (BM) test [48] was applied. Briefly, for each trial mice were placed under a transparent cylinder on a round circular platform with 20 equally distributed holes. One hole contained an escape box at a fixed location for all trials. Four different visual clues were placed around the platform. Bright light was used to induce escape behavior to the escape hole. Total duration of the BM test was 4 days, starting 4 days after the OF. Maximum test time per trial was 3 min. On the first day, animals were habituated to the arena in two trials. If the mice did not reach or enter the escape box they were carefully guided to and placed into the box. The 2nd and 3rd day of testing served as training days, to assess learning behavior. To measure spatial reference memory, the probe trial was performed on the 4th day. During this trial, the escape box was removed and all holes closed. The time animals needed to reach the trained escape hole was quantified.

### Statistical analysis

Results (if not indicated otherwise) are expressed as violin plots with individual data points including median values, the 25% and the 75% percentile. For statistical analysis, the GraphPad Prism 9.0 software package (GraphPad Software) was used. Data were tested for Gaussian distribution and analyzed either by ordinal one-way ANOVA (parametric) or by Kruskal–Wallis (non-parametric) with post hoc Sidak’s or Dunn’s multiple comparison tests, respectively. Data of BM testing were analyzed by 2-way-repeated measure ANOVA followed by Tukey’s multiple comparison test. Exploratory analyses of a potential impact of sex was determined with two-way ANOVA, with one factor sex and the other factor treatment (sham, NT, HT, HT + EV) followed by post hoc Sidak’s multiple comparisons test. Graphical presentation of data was performed, whenever significant main effects for sex or interaction between sex and treatment were detected.

## Results

### Intranasal ciMSC-EV therapy improves limited effects of acute HT on long-term HI-induced neurodevelopmental deficits

**Fig. 1:**
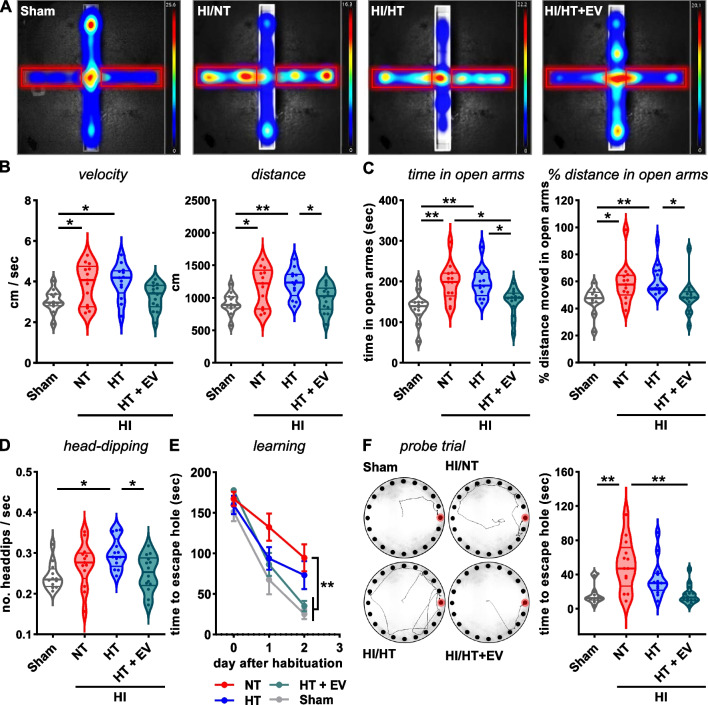
Combination of HT with intranasal ciMSC-EV treatment, but not HT monotherapy improves long-term functional deficits. Postnatal day 9 (P9) C57BL/6 mice were exposed to HI followed by 4 h NT or HT. Repetitive intranasal ciMSC-EV administration was performed at day 1, 3 and 5 after HI. Behavior was evaluated starting 35 days after HI with the Elevated Plus Maze (EPM) test. Representative heat maps of the time spent in different regions of the EPM are shown for each experimental group (**A**, red rectangles depict open arms). Mean velocities and total distances (**B**), the time and percentage of distance from the total distance moved (**C**) in the open arms of the EPM was quantified. Risk assessment behavior was evaluated by analyses of the frequency of head dippings in the open arms of the EPM (**D**). From day 40 after HI onwards cognitive function was assessed in the Barnes Maze (BM) test, measuring the time needed to enter the escape hole (**E**, d0 = day of habituation, d1 and d2 training days). On the 4th day spatial reference memory was analyzed in the probe trial, with all holes (including escape hole) closed (**F**, left: running pattern (escape hole in red), right: time needed to reach the escape hole, mean ± SEM). *n* = 12 (sham), *n* = 13 (NT), *n* = 13 (HT), *n* = 14 (HT + EV), *p < 0.05, **p < 0.01, ***p < 0.001. HI = hypoxia–ischemia, NT = normothermia/vehicle, HT = hypothermia/vehicle, HT + EV = hypothermia/ciMSC-EV

While HT significantly protects form death and severe disability [3], clinical findings revealed only limited improvement of long-term neuropsychological and cognitive deficits [2, 4–7, 9, 19]. In this context, we assessed activity and anxiety-related behavior in the Elevated Plus Maze (EPM) and Open Field (OF) test, followed by evaluation of spatial reference learning and memory function in the Barnes Maze (BM) test. Assessment of general activity in the EPM (Fig. 1A) 5 weeks after HI demonstrated that HI-injured animals move faster than healthy sham-operated mice, revealed by significantly increased mean velocities and moved distances during the 5 min testing phase (Fig. 1B). Furthermore, neonatal HI led to an increased time, the mice spent in the open arms of the maze (Fig. 1A, C). To exclude confounding effects by overall increased movement behavior on the time interval spent in the open arms, we further quantified the percentage of distances moved in the open arms from the total distances, confirming that HI-injured animals move more in the open arms (Fig. 1C). In addition to increased open arm activities, increased frequency of head dipping was previously associated with reduced anxiety [49–51]. Compared to sham-operated control mice, head dipping in the central platform and open arms of the maze were increased in HI animals (Fig. 1D). Importantly, while activity (Fig. 1A), movement in open arms (Fig. 1B, C) and head dipping (Fig. 1D) were similarly increased in HT-treated mice, additional intranasal ciMSC-EV therapy significantly reduced these long-term HI-induced deficits by 60–95% (Fig. 1A–D). HI-induced hyperactivity was also observed in the OF test, which was not altered by HT, but reduced to the level of sham animals by additional ciMSC-EV therapy (Additional file 1: Fig. S2A,B). In spite of a slightly increased time spent in the center region of the OF in HI-injured animals, no significant group differences were detected (Additional file 1: Fig. S2C). Learning capabilities, assessed in the BM, were significantly reduced in HI-injured animals, demonstrated by a 4 × longer time needed to reach the trained escape hole on the 3rd day of testing (Fig. 1E). This deficit could not be restored by HT monotherapy but by the combined therapy with ciMSC-EVs (Fig. 1E). In the spatial probe test as an indicator for adaptive memory function performed at the fourth day, HI-injured animals exposed to HT/ciMSC-EV treatment but not to a HT/vehicle treatment needed significantly less time, comparable to sham animals to reach the trained escape hole (Fig. 1F).

### Combined HT/ciMSC-EV treatment but not HT monotherapy protects from HI-induced neuronal and axonal injury

**Fig. 2:**
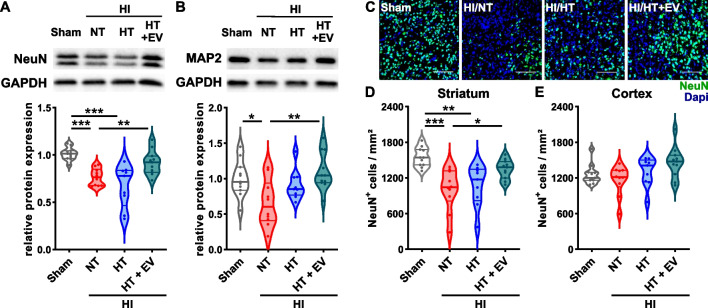
Intranasal ciMSC-EV therapy overcomes limited effects of HT on secondary HI-induced neurodegeneration in the striatum. C57BL/6 mice were exposed to HI on postnatal day 9 (P9) followed by 4 h HT or NT. ciMSC-EVs were delivered intranasally on day 1, 3 and 5 after HI. Western blot analyses with tissue lysates of ipsilateral hemispheres from 160-µm-thick tissue sections derived from the striatal level were performed for NeuN (**A**) and MAP2 (**B**) 7 days after HI. Data were normalized to the reference protein GAPDH and to sham animals. The number of neurons positively stained for NeuN (**C**) was quantified via immunohistochemistry in the striatum (**D**) and cortex (**E**). Representative images in **A** and **B** were cropped and scaled from original full length western blots provided in Additional file 1: Fig. S4. Representative images in **C** are derived from the striatum (scale bar 100 µm). *n* = 11 (sham), *n* = 10 (NT), *n* = 9 (HT), *n* = 11 (HT + EV), *p < 0.05, **p < 0.01, ***p < 0.001. HI = hypoxia–ischemia, NT = normothermia/vehicle, HT = hypothermia/vehicle, HT + EV = hypothermia/ciMSC-EV

To get deeper insight into the underlying protective mechanisms of the combination therapy, we performed in-depth analyses of brain tissues 7 days after HI. According to our previous work [15, 18, 27, 31], revealing most pronounced effects of an ciMSC-EV treatment and limited protection of HT in the striatum, we focused our analyses on this particular brain level. Neuronal and axonal injury, analyzed via western blot for NeuN (Fig. 2A) and MAP2 (Fig. 2B), respectively, showed a strong decrease for both proteins in NT/vehicle HI-injured mice. While HT monotherapy slightly increased MAP2 expression, no differences were detected for NeuN (Fig. 2A, B). However, the combined treatment with ciMSC-EVs increased both, NeuN and MAP2 expression, comparable to healthy sham animals (Fig. 2A, B). Regional analyses of neuronal loss via immunohistochemistry (Fig. 2C) revealed that effects observed in tissue lysates of the total hemisphere at the striatal level (Fig. 2A, B), were mainly caused by a prominent reduction of striatal neurons in both, NT/vehicle- and HT/vehicle-, but not in HT/ciMSC-EV-treated animals (Fig. 2D) No group differences were observed in the cortex (Fig. 2E).

### HT and HT/ciMSC-EV therapy similarly protect from HI-induced vascular injury, while effects on endothelial proliferation are more pronounced in the combined treatment

**Fig. 3:**
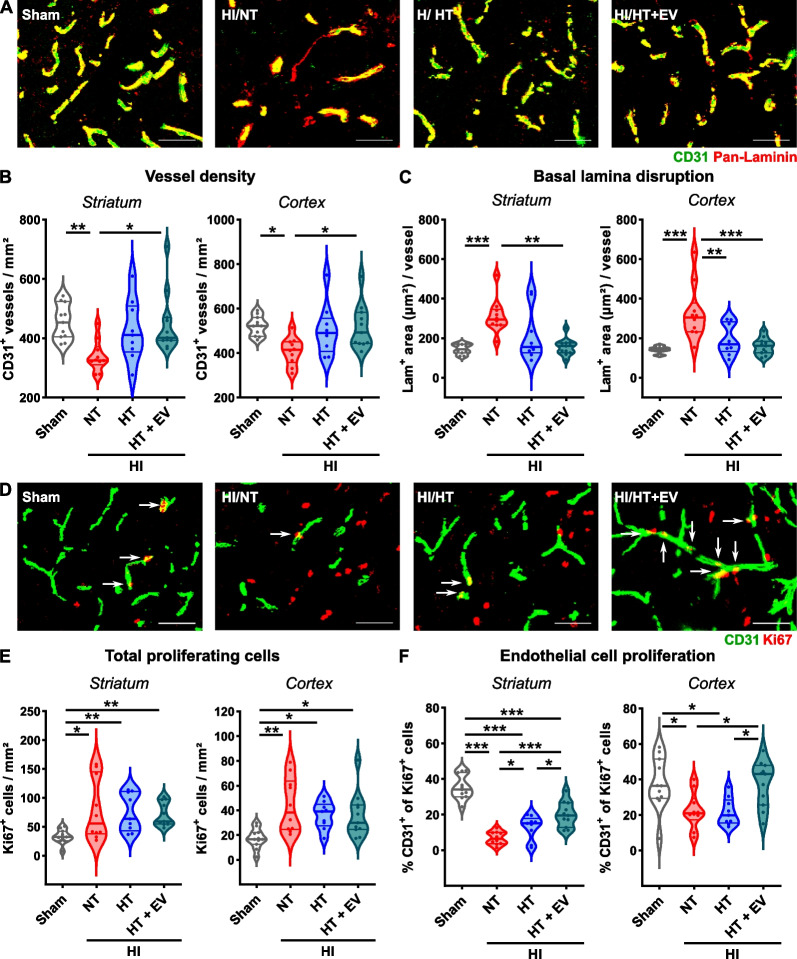
HT combined with ciMSC-EV treatment protects from vascular injury and promotes endothelial cell proliferation. Vessel densities, vascular injury and endothelial proliferation were determined in the cortex and striatum of postnatal day 16 (P16) C57BL/6 mice subjected to HI on P9, followed by 4 h NT or HT. Repetitive intranasal ciMSC-EV delivery was performed on day 1, 3 and 5 after HI. Vascular density and injury were determined in CD31 (green) and pan-Laminin (red)-stained tissue sections (**A**, scale bar: 50 µm) at day 7 after HI. Vessel densities (**B**) and endothelial basal lamina disruption (**C**) were quantified in the striatum and cortex. Total and endothelial cell proliferation was analyzed via immunohistochemistry for CD31 (green) and the proliferation marker Ki67 (red) in the striatum and cortex (**D**, arrows indicate CD31/Ki67 double positive cells, scale bar: 50 µm). Representative images in **A** and **D** are derived from the striatum. *n* = 11 (sham), *n* = 10 (NT), *n* = 9 (HT), *n* = 11 (HT + EV), *p < 0.05, **p < 0.01, ***p < 0.001. HI = hypoxia–ischemia, NT = normothermia/vehicle, HT = hypothermia/vehicle, HT + EV = hypothermia/ciMSC-EV

In addition to neuronal injury, HI induces vascular damage, demonstrated by a pronounced vessel loss and dispersal of normally tight basement membranes (Fig. 3A). Both, HT monotherapy and the combined treatment with ciMSC-EVs, reduced HI-induced vessel loss in the striatum and cortex, though significant differences were only detected for the combined therapy (Fig. 3B). Basal lamina disruption was also mitigated by both interventions, demonstrated by a reduced laminin-positive area reaching median levels of healthy sham-operated animals (Fig. 3C). To compensate injury to neurons and vessels, HI induces an endogenous proliferative response, reflected by an increased number of Ki67^+^ cells, which was not modulated by either therapy (Fig. 3D, E). However, the proportion of proliferating endothelial cells in the striatum and cortex of HI-injured animals was strongly decreased (Fig. 3D, F). Though HT led to a significant increase in the striatum compared to NT/vehicle-treatment, no effects were determined in the cortex (Fig. 3F). Importantly, combined treatment with ciMSC-EVs was more effective, revealed by a significantly increased proportion of proliferating endothelial cells compared to HT monotherapy in striatum and cortex (Fig. 3D, F).

### The combined therapy with HT and intranasal ciMSC-EVs prevents endothelial activation and leukocyte infiltration

**Fig. 4:**
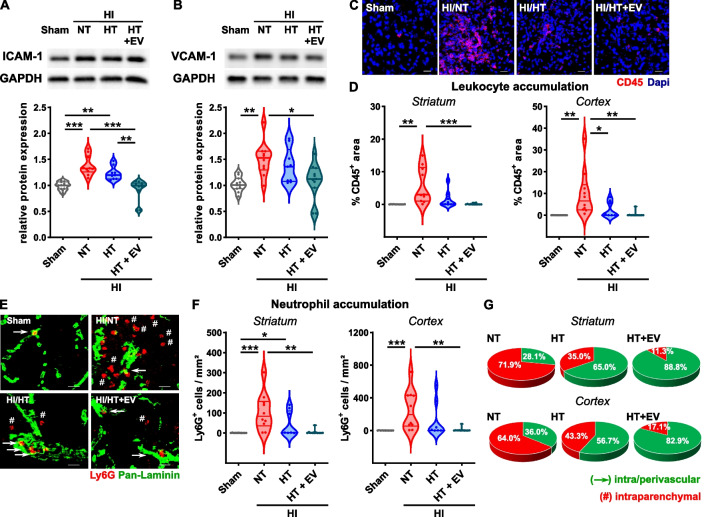
HT combined with ciMSC-EV application reduces HI-induced endothelial activation and leukocyte accumulation. C57BL/6 mice were exposed to HI on postnatal day 9 (P9) followed by 4 h HT or NT. ciMSC-EVs were delivered intranasally on day 1, 3 and 5 after HI. Endothelial activation was assessed via western blot analyses for the adhesion molecules ICAM-1 (**A**) and VCAM-1 (**B**) in whole hemisphere protein lysates derived from 160-µm-thick tissue sections at the striatal level at day 7 after HI. Data were normalized to the reference protein GAPDH and to sham animals. Leukocyte accumulation was determined in CD45-stained tissue sections (**C**, scale bar: 20 µm). CD45 positively stained areas were quantified in the striatum and in the cortex (**D**). Neutrophil accumulation and localization was analyzed via immunohistochemistry in sections stained for Ly6G (red) and pan-Laminin (green) (**E** arrows indicate Ly6G neutrophils in the intra/perivascular space, rhombi indicate intraparenchymal neutrophils, scale bar: 20 µm). Neutrophil accumulation was quantified by counting Ly6G positive cells in the striatum and cortex (**F**) and the percentage of intraparenchymal and intra/perivascular neutrophils was quantified in HI-injured animals (**G**). Representative images in A and B were cropped and scaled from original full length western blots provided in Additional file 1: Fig. S4. Representative images in **C** and **E** are derived from the striatum *n* = 11 (sham), *n* = 10 (NT), *n* = 9 (HT), *n* = 11 (HT + EV), *p < 0.05, **p < 0.01, ***p < 0.001. HI = hypoxia–ischemia, NT = normothermia/vehicle, HT = hypothermia/vehicle, HT + EV = hypothermia/ciMSC-EV

Besides injury to the vasculature, HI induces endothelial activation, which in addition to vessel disruption facilitates accumulation of peripheral leukocytes in the brain. HI-induced endothelial activation characterized by an increased expression of the adhesion molecules ICAM-1 and VCAM-1 was significantly reduced in HT/ciMSC-EV- but not in HT/vehicle-treated mice (Fig. 4A, B). Analyses of leukocyte accumulation via immunohistochemistry revealed a marked reduction of leukocytes in both therapeutic intervention groups, though effects in the combined treatment setting were more pronounced (Fig. 4C, D). According to previous work in adult ischemic brain injury, suggesting neutrophils to be a specific target of MSC-EVs [52], we further analyzed the amount of accumulated neutrophils demonstrating a similar regulation (Fig. 4E, F). The association between both, total leukocyte and neutrophil accumulation, was confirmed by a significant correlation (Additional file 1: Fig. S3). In view of discussions whether neutrophils infiltrate deeply into the injured brain parenchyma or rather stick to the perivascular space [53, 54], we combined anti-Ly6G staining with laminin-staining (Fig. 4E) to quantify the proportion of peri-/intravascular and intra-parenchymal neutrophils (Fig. 4G). Analyses were performed only in HI-injured animals, because we hardly detected any neutrophils in brains of sham animals. While the majority of cells in NT/vehicle-treated mice was found deep in the parenchyma, approximately 60% to 80% of neutrophils were found either in the vessels or in the perivascular space of HT/vehicle- and HT/ciMSC-EV-treated mice (Fig. 4G).

### HT/ciMSC-EV treatment, but not HT monotherapy reduces HI-induced microglia activation, astrogliosis and pro-inflammatory cytokine expression, while expression of anti-inflammatory cytokines and trophic growth factors is increased

**Fig. 5:**
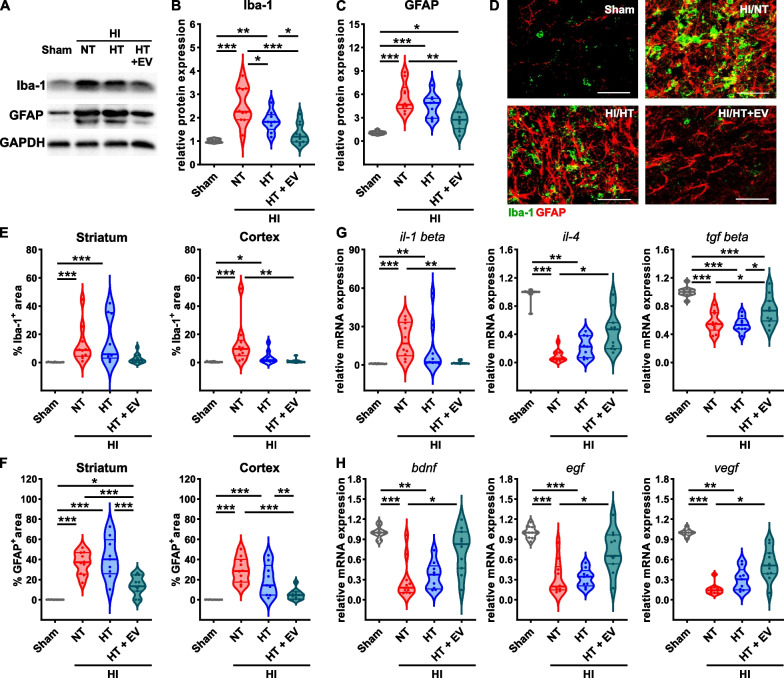
ciMSC-EV treatment mitigates limitations of HT on secondary neuroinflammation and enhances neurotrophic growth factor expression. HI-induced brain injury was induced in postnatal day 9 (P9) mice, followed by 4 h NT or HT. Intranasal ciMSC-EV administration was performed 1, 3 and 5 days after HI followed by western blot, immunohistochemistry and real-time PCR analyses 7 days after HI. Western blot analyses for Iba-1 and GFAP (**A**) were performed in tissue lysates of the entire hemisphere derived from 160 µm thick tissue sections at the striatal level to quantify microglia (**B**) and astrocyte (**C**) activation. Data were normalized to the reference protein GAPDH and sham animals. Immunohistochemistry was performed for Iba1 (green) and GFAP (red) (**D**, scale bar: 100 µm). Microglia (**E**) and astrocyte (**F**) activation were analysed by quantification of positively stained areas in the cortex and striatum. The expression of pro- and anti-inflammatory cytokines (**G**) and neurotrophic growth factors (**H**) was measured via real-time PCR in brain tissue lysates of the entire hemisphere derived from 160 µm thick tissue sections at the striatal level. Beta-2-microglobulin served as a housekeeping gene and fold change values were calculated compared to sham animals. Representative images in **A** were cropped and scaled from original full length western blots provided in Additional file 1: Fig. S4. Representative images in **D** are derived from the striatum. *n* = 11 (sham), *n* = 10 (NT), *n* = 9 (HT), *n* = 11 (HT + EV), *p < 0.05, **p < 0.01, ***p < 0.001. HI = hypoxia–ischemia, NT = normothermia/vehicle, HT = hypothermia/vehicle, HT + EV = hypothermia/ciMSC-EV

Concomitant with the accumulation of peripheral immune cells in the injured brain, HI elicits strong inflammatory responses in brain resident cells, i.e., microglia and astrocytes. Activation of both cell types can be measured by an increased protein abundance of Iba-1 and GFAP, respectively [55, 56]. Western blot analyses of Iba-1 and GFAP expression in tissue lysates of the entire hemisphere at the level of the striatum, demonstrated a strong increase in NT-treated HI-injured mice, which was not altered by HT monotherapy (Fig. 5A–C). In contrast, the combined treatment with ciMSC-EVs resulted in a significant decrease of Iba-1 and GFAP expression by 85 and 40%, respectively (Fig. 5A–C). Similar findings were obtained from immunohistochemistry analyses in the striatum and cortex (Fig. 5D–F). To characterize immunomodulatory effects at the molecular level, we further investigated expression of pro- and anti-inflammatory cytokines via real-time PCR. Congruent with results for Iba-1 and GFAP expression, we detected a strong upregulation of the pro-inflammatory cytokine interleukin (IL)-1 beta and a pronounced reduction of the anti-inflammatory cytokines IL-4 and transforming growth factor (TGF)-beta in NT/vehicle and HT/vehicle-treated HI-injured mice compared to healthy sham-operated animals (Fig. 5G). Importantly, these effects were counteracted by the combined therapy with HT and ciMSC-EVs (Fig. 5G). In addition to production of inflammatory mediators, glial cells, particularly astrocytes, are main sources of trophic growth factors, contributing to regeneration and repair [57]. In accordance with our previous work [27, 31], HI led to a marked reduction of brain derived nerve factor (BDNF), epidermal growth factor (EGF) and vascular endothelial growth factor (VEGF) by approximately 80% (Fig. 5H). While HT monotherapy treatment had no impact, the combination with an intranasal ciMSC-EV therapy significantly attenuated this HI-induced deficit (Fig. 5H).

### The combined treatment with HT and ciMSC-EVs, but not HT alone improves HI-induced myelination deficits

**Fig. 6:**
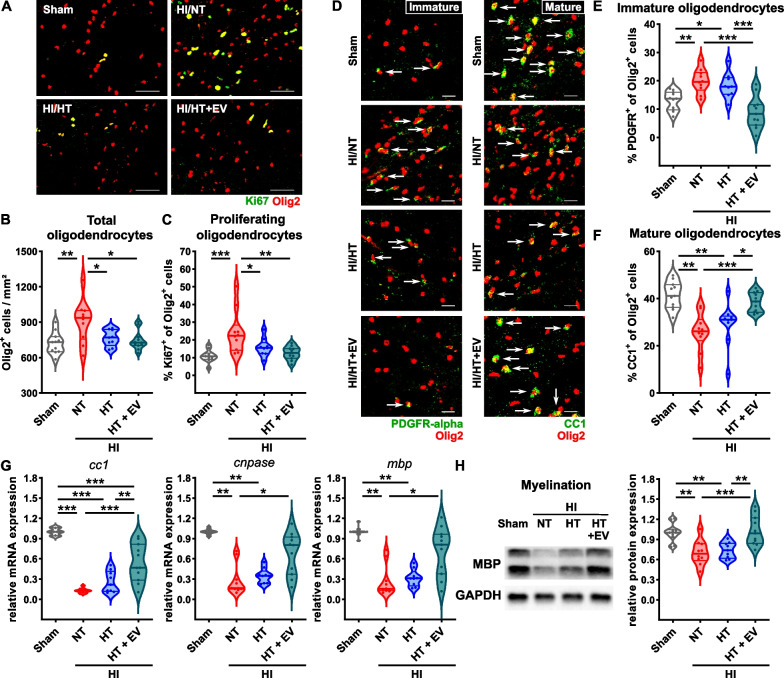
Intranasal ciMSC-EV delivery overcomes limitations of HT on HI-induced impaired oligodendrocyte maturation and myelination. Postnatal day 9 (P9) mice were exposed to HI followed by 4 h NT or HT. Intranasal ciMSC-EV administration was performed 1, 3 and 5 days after HI. Immunohistochemistry, real-time PCR and western blot analyses were performed 7 days after HI. Oligodendrocyte proliferation was assessed in Olig2/Ki67 double stained tissue sections (**A**, Olig2 (red), Ki67 (green), scale bar: 50 µm). The total amount of oligodendrocytes (**B**) and proportion of proliferating oligodendrocytes (**C**) was quantified. Oligodendrocyte maturation was analyzed via immunohistochemistry through co-staining of Olig2 with either PDGFR alpha for immature oligodendrocytes (**D**, left) or CC1 for mature oligodendrocytes (**D**, right), each indicated by arrows (**D**, scale bar: 20 µm). The proportion of Olig2/PDGFR alpha (**E**) and CC1/Olig2 (**F**) double-positive cells was quantified. Real-time PCR (**G**) and western blot analyses (**H**) were performed in tissue lysates derived from ipsilateral hemispheres of 160 µm thick tissue sections at the striatal level for markers expressed by mature and myelinating oligodendrocytes. For real-time PCR, beta-2-microglobulin served as a housekeeping gene and fold change values were calculated compared to sham animals (**G**). For western blot analysis data were normalized to the reference protein GAPDH and sham animals. Representative images in **A** and **D** are derived from the external capsule of the white matter. Representative images in **H** were cropped and scaled from original full length western blots provided in Additional file 1: Fig. S4. *n* = 11 (sham), *n* = 10 (NT), *n* = 9 (HT), *n* = 11 (HT + EV), *p < 0.05, **p < 0.01, ***p < 0.001. HI = hypoxia–ischemia, NT = normothermia/vehicle, HT = hypothermia/vehicle, HT + EV = hypothermia/ciMSC-EV

While HI-induced inflammatory responses contribute to secondary neurodegeneration, endogenous regenerative responses, e.g., oligodendrocyte proliferation are initiated [15, 18]. However, previous work suggested that these newly generated cells do not differentiate into mature myelinating oligodendrocytes [15, 18, 27, 31]. To assess the proportion of proliferating, immature and mature oligodendrocytes, we performed immunohistochemistry analyses for the pan-oligodendrocyte marker Olig2 stained in combination with either Ki67 or PDGFR-alpha or CC1, respectively. HI-induced proliferation, revealed by an increased total number and an increased proportion of Ki67^+^ oligodendrocytes, was reduced by both, HT/vehicle and HT/ciMSC-EV treatment (Fig. 6A–C). Interestingly, the proportion of immature oligodendrocytes was increased (Fig. 6D, E) while the amount of mature oligodendrocytes was reduced following HI (Fig. 6D, F). While HT monotherapy could not restore this maturation deficit, the combined treatment with ciMSC-EVs resulted in a significantly elevated proportion of mature oligodendrocytes with a concomitant decrease of immature oligodendrocytes (Fig. 6D–F). To confirm effects on oligodendrocyte maturation, we quantified mRNA expression of CC1 and the myelin proteins CNPase and MBP, demonstrating a strong downregulation of these molecules, which was attenuated by HT/ciMSC-EV therapy but not by HT/vehicle treatment (Fig. 6G). Western blot analyses of MBP confirmed improvement of myelination deficits by the combined treatment with HT and ciMSC-EVs (Fig. 6H).

## Discussion

Large clinical multicenter trials have proven beneficial effects of HT in reducing death or severe disability following neonatal HI-induced brain injury [2, 58]. However, an unacceptably high proportion of children still suffer from adverse long-term neurological outcomes, especially including cognitive and emotional deficits [2]. Using a translational rodent model of HI and HT, we demonstrate that additional intranasal delivery of ciMSC-EVs overcomes limitations of HT on HI-induced neurodevelopmental deficits. Long-term protection from functional deficits was preceded by a significant reduction of HI-induced neuronal loss in the striatum. Although HT prevented vascular injury, the combined therapy improved deficits of HT on endothelial proliferation, neurotrophic growth factor expression and oligodendrocyte maturation. Enhanced regenerative responses were accompanied by stronger anti-inflammatory effects, demonstrated by reduced endothelial, microglia and astrocyte activation as well as decreased expression of pro-inflammatory and increased expression of anti-inflammatory cytokines.

The most important finding of the present study is that combination with ciMSC-EV therapy mitigates limitations of HT on long-term cognitive deficits and HI-induced alterations in anxiety-related behavior. Limited effects of HT monotherapy on cognitive dysfunction demonstrated in the present and previous experimental studies [15, 18, 59] are supported by clinical observations, i.e., cooled HI-injured infants frequently develop difficulties in learning and visual-spatial processing at school age and adolescence [2, 4, 5, 7–9]. While the majority of experimental studies, investigating MSC-EVs in neonatal HI-induced brain injury focussed on short-term outcomes [27, 28, 31, 32], we show that intranasal ciMSC-EV therapy overcomes limitations of HT on long-term learning and memory difficulties. In addition to cognitive deficits, persisting alterations in emotional behavior, e.g., hyperactivity and attention dysfunctions have been described in HT-treated infants [2, 4, 5, 9]. In the present experimental study with mice, we observed that HI-injured animals move with higher velocity and travel longer distances in the EPM and OF test, which may indicate signs of hyperactivity. Furthermore, alterations in anxiety-related behavior were demonstrated by more movement in the open arms of the EPM and higher frequencies of head dipping, both associated with reduced anxiety [49–51, 60]. We are aware about difficulties associated with direct translation of rodent behavior to humans, in which potential psycho-emotional impairments following a neonatal HI insult might be more complex, not only involving a single behavioral trait, like anxiety or hyperactivity, but probably an interdependent combination with other difficulties like conduct problems and impaired social skills [4, 5]. To mimic these complex behavioral alterations assessment of additional behavioral tasks, e.g., social interaction tests would be needed. Nevertheless, increased activity and reduced anxiety at least reflect two symptoms of psychological alterations following HI. Importantly, these HI-induced behavioral changes were not modulated by immediate HT monotherapy, while additional intranasal ciMSC-EV treatment starting one day after HI/HT improved these long-term HI-induced deficits. These findings support the concept of different windows of opportunities for therapeutic interventions in neonatal HI. Acute HT is supposed to target early pathophysiological mechanisms, which may not compensate or only delay secondary injury processes, not altering endogenous regenerative and/or disturbed physiological neurodevelopmental processes [10, 11, 61].

Protection by HT is limited to certain brain structures. Previous work showed that an immediate HT leads to neuroprotection in the hippocampus with little to no effect in the striatum [13–15, 62]. We also did not detect protection from striatal neuronal loss by HT. Differences in cell death mechanisms but also different time windows of neuronal degeneration may account for these regional differences. As such, acute HT does not protect from loss of medium spiny neurons, covering 95% of the striatum [14], while in the same experimental setting continuous infusion of the neurotrophic growth factor BDNF for 3 days after injury protected from HI-induced loss of striatal neurons [63]. Interestingly, ciMSC-EV therapy led to an increased expression of important growth factors, including BDNF, which might have contributed to increased survival of striatal neurons in this and our recent study [31]. Striatal protection appears particularly important for long-term neurodevelopment as revealed by associations between basal ganglia injury and adverse neurological outcome [64, 65]. While basal ganglia injury was predominantly associated with motor function deficits, new concepts suggest that the basal ganglia also have major functions in relation to learning habits, since striatal circuitry not only generalizes to the motor domain, but also to cognitive skills and emotion-related behaviors [66]. Therefore, the pronounced protection in the striatum after additional ciMSC-EV therapy may have contributed to improvement of long-lasting cognitive deficits and changes in activity and anxiety compared to HT monotherapy. Besides limited effects of HT on striatal injury, clinical and pre-clinical reports suggest that white matter injury is only partially restored leading to persisting myelination deficits [15–20, 27, 31], which are associated with lower learning and personal–social–emotional skills [17, 20, 21]. The present work confirmed these findings, as revealed by a lack of protection from HI-induced myelination deficits in HT/vehicle-treated mice. Although HI induces an endogenous proliferative response to compensate for oligodendrocyte injury, these newly generated cells apparently do not differentiate into mature myelin-forming oligodendrocytes [15, 18, 27, 31]. In the present study, we show that improved long-term neurological outcome in the combined setting of HT/ciMSC-EV therapy was associated with improved oligodendrocyte maturation and myelination, which might explain improved long-term neurodevelopmental outcome in the combination therapy compared to HT monotherapy.

An ideal adjunct therapy to HT should target mechanisms of the secondary and tertiary disease phase, promoting regeneration, but also enhancing early protective and anti-inflammatory effects of HT [10, 11]. HT monotherapy and its combination with ciMSC-EVs prevented vascular injury, resulting in a decreased accumulation of peripheral immune cells and specifically neutrophils, most likely caused by a similar amelioration of vascular damage (i.e., basal lamina disruption). Nevertheless, we detected more pronounced protective effects on total leukocyte/neutrophil accumulation and intra-/perivascular localization of neutrophils after combined treatment. This might be related to additional effects of ciMSC-EVs on endothelial activation, i.e., ICAM-1 and VCAM-1 expression on remaining intact vessels, facilitating leukocyte transmigration independent of vascular damage. With regard to inflammatory responses in brain resident cells (i.e. microgliaglia and astrocytes), we detected increased anti-inflammatory effects in the combined treatment setting compared to HT monotherapy. In accordance with previous work [15], HT did not modulate astrogliosis. Regarding microglia activation, we recently showed that HT significantly reduced expression of pro-inflammatory cytokines 24 h after the insult [42]. However, HT had limited effects on delayed inflammatory responses in microglia, e.g., expression of the anti-inflammatory cytokine TGF-beta was similarly reduced in microglia of HT- and NT-treated mice 7 days after HI [42]. Our present results from total tissue lysates are in good agreement with this, since HT monotherapy could not restore HI-induced reduction of IL-4 and TGF-beta expression. However, the additional treatment with ciMSC-EVs boosted limited anti-inflammatory effects of HT, revealed by decreased microglia and astrocyte activation, reduced IL-1 beta and enhanced IL-4 and TGF-beta expression compared to HT monotherapy. Keeping the delayed treatment regime with ciMSC-EVs until 5 days after HI in mind, delayed ciMSC-EV treatment apparently fosters HT’s initial anti-inflammatory effects to inhibit ongoing inflammation in the subacute disease phase, thereby facilitating an environment for improved regeneration. Indeed, while HT single treatment did not prevent the strong HI-induced reduction of trophic growth factors, additional ciMSC-EV treatment resulted in a significantly increased expression of BDNF, EGF and VEGF. These neurotrophic factors are important molecular components in the regenerative response, but also during neurodevelopment, supporting oligodendrocyte maturation and angiogenesis [67–70]. In line with that, HT combined with a ciMSC-EV treatment led to an increased endothelial proliferation and oligodendrocyte maturation compared to HT monotherapy.

Previous pre-clinical and clinical studies suggested pronounced sex differences in outcome and therapy responses following perinatal brain injury [45, 71–73]. Descriptive analyses of potential sex differences in the present work revealed similar responses to injury and therapies in both, females and males for the majority of outcome measures (Additional file 1: Table S5-8). However, results have to be interpreted with caution, since sex-stratified analyses were not the primary aim of the present study and, therefore, sample sizes are limited to draw final clear-cut conclusions due to the well-known variability in this injury model. This is supported by the fact, that despite significant main treatment effects obtained from two-way ANOVA analyses (Additional file 1: Table S5,6, highlighted in blue italics), post hoc testing with correction for multiple comparisons often did not result in significant group differences in each sex (Additional file 1: Table S7,8). Nevertheless, for a few outcomes significant main effects of sex or interaction between sex and treatment were detected (Additional file 1: Table S5,6, highlighted in red bold). Sex differences became evident at the physiological level, but also in response to the insult and the investigated therapies. Basal sex differences were observed for learning capabilities, i.e., compared to sham-operated males, females needed 40% less time to enter the trained escape hole in the BM test at the first day after habituation (Additional file 1: Fig. S5A). To date, sex differences in learning traits are not fully understood, depending on several factors, e.g., the test paradigm and species [74, 75]. For instance, in the water maze test, male rats showed an advantage over females, while in mouse studies females performed slightly better than males [74], supporting the present findings. Physiological sex differences were also presented by a reduced percentage of proliferating endothelial cells by 25% and 40% in the striatum and cortex, respectively, in sham-operated males compared to females (Additional file 1: Fig. S5B). Though mainly described in adults and related to hormonal influences [76], intrinsic physiological sex differences in endothelial cells were recently also documented at birth [77]. In addition to basal differences, we detected sex-differences in response to the HI-insult, reflected by an overall increased proliferative response in the cortex (Additional file 1: Fig. S5C) and specifically a significantly enhanced proportion of proliferating oligodendrocytes in the white matter by almost 50% in HI-injured male mice compared to females (Additional file 1: Fig. S5D). Previous studies suggested opposite effects, which were, however, mainly described in in vitro studies or in the context of brain aging and related pathology [78, 79]. With regard to perinatal brain injury, far less is known and requires further investigation. Besides different responses to the HI-insult itself, different therapy responses have been observed. HT/ciMSC-EV therapy improved myelination deficits to levels of sham animals only in females (Additional file 1: Fig. S5E). Similar enhanced responses to the combined therapy in females were observed for the percentage of proliferating endothelial cells (Additional file 1: Fig. S5B) and HI-induced astrogliosis (Additional file 1: Fig. S5F). In addition to improved treatment effects in the combined therapy setting, a better response to HT monotherapy was determined for vascular density in the striatum (Additional file 1: Fig. S5G). Together, these findings support previous experimental studies, suggesting better treatment responses to different therapeutic interventions in females [80–82]. However, besides small sample sizes, most of the identified sex differences were not consistently detected in all brain regions (Additional file 1: Table S5-S8, Additional file 1: Fig. S5), adding another level of complexity to be considered.

## Conclusion

Taken together, delayed intranasal delivery of ciMSC-EVs overcomes limited effects of acute HT on HI-induced striatal and white matter injury through boosting regenerative and anti-inflammatory effects in the secondary and tertiary disease phase, culminating in an improved long-term neurodevelopmental outcome. A particular strength of the present study is the assessment of long-lasting behavioral changes. We identified HI-induced cognitive deficits and alterations in anxiety/activity-related behavior, which were significantly improved by combined HT/ciMSC-EV treatment. However, differences in mouse and human behavior and the limited number of behavior tests, applied in the present study should be considered. Further studies applying a broader set of behavioral tests targeting particularly psychological traits are needed to increase the translational value of the present findings. We administered EVs from clonally expanded immortalized MSCs, overcoming limitations associated with MSCs’ plasticity, heterogeneity and senescence. The possibility of intranasal delivery increases the translational potential of the suggested therapy. However, according to anatomical and physiological differences of the nasal cavity between rodents and humans, further proof-of-principle studies in large animal models exploring i.n. delivery of stem cell-based therapies in perinatal brain injury are needed. Furthermore, since sex-stratified analyses were not the primary aim of the present work, samples sizes were limited to draw clear-cut conclusions about the impact of sex. Nevertheless, our descriptive analyses provided first interesting insights into sex-specific responses to the HI-insult but also the investigated therapies, as a potential basis for future clinical and pre-clinical trials.

We performed in-depth analyses of secondary injury processes, including inflammatory, regenerative and neurodevelopmental responses to clearly delineate limitations of HT from an additional benefit that can be provided by ciMSC-EVs. A single ciMSC-EV therapy was not included in the present study, because we applied the same dose and treatment protocol, using the same EV preparation as in our recent work [31]. Adhering to the 3R principle of animal welfare, we decided not to repeat these in vivo experiments with a ciMSC-EV monotherapy. Similar outcome measures in our previous report [31] allowed comparisons between the present and our former results. From these comparisons, it becomes evident that most of the protective effects of a ciMSC-EV single treatment [31] were confirmed in the combined treatment with HT, suggesting that ciMSC-EVs have additive instead of synergistic effects. Therefore, ciMSC-EVs may not only be an adjunct therapy for HT, but also a standalone therapy in settings when HT cannot be applied or is ineffective, e.g., in middle- and low-income countries, where HT has shown little to no protection or even detrimental effects [83, 84].

### Supplementary Information

**Additional file 1.** Supplementary figures and tables.

## Data Availability

All data supporting the findings of this study are available in the main figures with individual data points presented and in the supplementary material of this article.

## Abbreviation

MSC: Mesenchymal stem/stromal cell
EV: Extracellular vesicle
ciMSC: Clonally expanded immortalized MSC
HI: Hypoxia–ischemia
HT: Hypothermia
NT: Normothermia
NE: Neonatal encephalopathy
NeuN: Neuronal nuclei
Olig-2: Oligodendrocyte transcription factor-2
CD: Cluster of differentiation
GFAP: Glial fibrillary acidic protein
Iba-1: Ionized calcium binding adaptor protein-1
Ly6G: Lymphocyte antigen 6 complex locus G6D
PDGFR-alpha: Platelet-derived growth factor receptor-alpha
APC-CC1: Adenomatous polyposis coli, clone CC1
PFA: Paraformaldehyde
Dapi: 4’,6-Diamidino-2-phenylindole
ROI: Region of interest
PCR: Polymerase chain reaction
MAP2: Microtubule associated protein 2
VCAM-1: Vascular cell adhesion molecule-1
ICAM-1: Intercellular adhesion molecule-1
MBP: Myelin basic protein
GAPDH: Glutaraldehyde-3-phosphate dehydrogenase
EPM: Elevated plus maze
OF: Open field
BM: Barnes maze
IL: Interleukin
TGF beta: Transforming growth factor beta
BDNF: Brain derived nerve factor
EGF: Epidermal growth factor
VEGF: Vascular endothelial growth factor
CNPase: 2’,3’-Cyclic nucleotide 3’-phosphodiesterase
