# Supplementary material for: Hypothermia combined with extracellular vesicles from clonally expanded immortalized mesenchymal stromal cells improves neurodevelopmental impairment in neonatal hypoxic-ischemic brain injury
Source: J Neuroinflammation. 2023 Nov 27;20:280. doi: 10.1186/s12974-023-02961-0 (PMC10680187; doi:10.1186/s12974-023-02961-0)
Supplement: Supplementary file 1 — Additional file 1. Supplementary figures and tables. [file 12974_2023_2961_MOESM1_ESM.pdf]

Additional File1: Supplementary Figures and Tables

Hypothermia combined with extracellular vesicles from clonally expanded immortalized mesenchymal stromal cells improves neurodevelopmental impairment in neonatal hypoxic-ischemic brain injury

Nicole Labusek[1], Parnian Ghari[1], Yanis Mouloud[2], Christian Köster[1], Eva Diesterbeck[1], Martin Hadamitzky[3], Ursula Felderhoff-Müser[1], Ivo Bendix[1], Bernd Giebel[2], Josephine Herz[1#]

[1]Department of Pediatrics I, Neonatology & Experimental perinatal Neurosciences, Center for Translational Neuro- and Behavioral Sciences (C-TNBS), University Hospital Essen, University Duisburg-Essen, Essen, Germany

[2]Institute for Transfusion Medicine, University Hospital Essen, University of Duisburg-Essen, Essen, Germany

[3]Institute for Medical Psychology, Center for Translational Neuro- and Behavioral Sciences (C-TNBS), University Hospital Essen, University of Duisburg-Essen, Essen, Germany

[#]Correspondence to:

Josephine Herz: josephine.herz@uk-essen.de

Supplementary Figures: **5**

Supplementary Tables **8**

Supplementary Figures

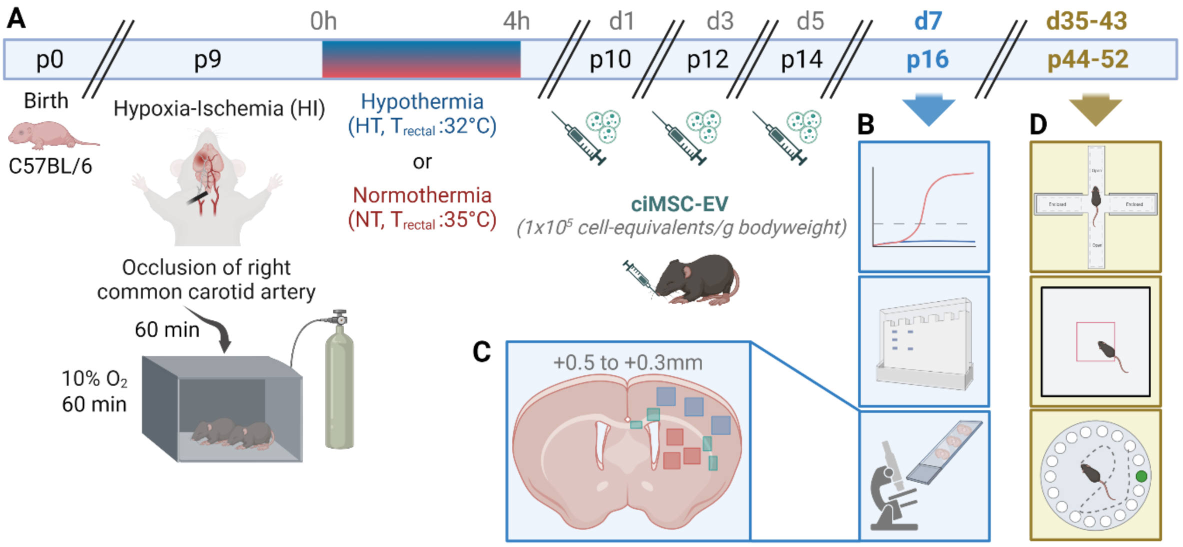

Figure S1: Experimental setup and readouts. Neonatal HI was induced in postnatal day 9 (P9) C57BL/6 mice followed by immediate HT or NT (**A**). ciMSC-EVs were administered intranasally at the indicated time points (**A**). One week after HI, brain injury, neuroinflammatory and regenerative responses were evaluated via real time PCR, western blot analyses and immunohistochemistry (**B**) at the level of the striatum in the indicated brain regions (**C**, red: striatum (3 ROIs: 600 x 600 µm), blue: cortex (3 ROIs: 650 x 650 µm), green: white matter (corpus callosum: 400 x 250 µm, cingulum: 450 x450 µm, 2 ROIs external capsule: 350 x 500 µm)). Five weeks after HI, behavioural testing was started with the Elevated Plus Maze, followed by the Open Field and the Barnes Maze test (**D**). The figure was created with biorender.com.

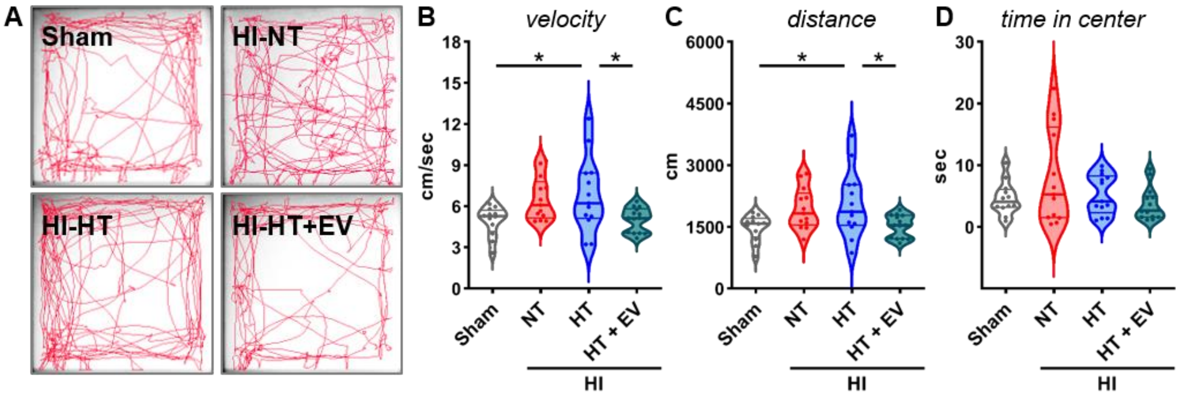

Figure S2: Combination of acute HT with intranasal ciMSC-EV treatment, but not HT monotherapy reduce HI-induced hyperactivity. Postnatal day 9 (P9) C57BL/6 mice were exposed to HI followed by 4 h NT or HT. Intranasal ciMSC-EV administration was performed at day 1, 3 and 5 after HI. Behavior was evaluated 35 days after HI in the Open Field test. Representative images of the running pattern for the test duration of 5 min are shown for each experimental group (**A**). Mean velocities (**B**), total distances (**C**) and the time moved in the center region (**D**) were quantified. n = 12 (sham), n = 13 (NT), n = 13 (HT), n = 14 (HT+EV), *p<0.05. HI = hypoxia-ischemia, NT = normothermia/vehicle, HT = hypothermia/vehicle, HT+EV = hypothermia/ciMSC-EV

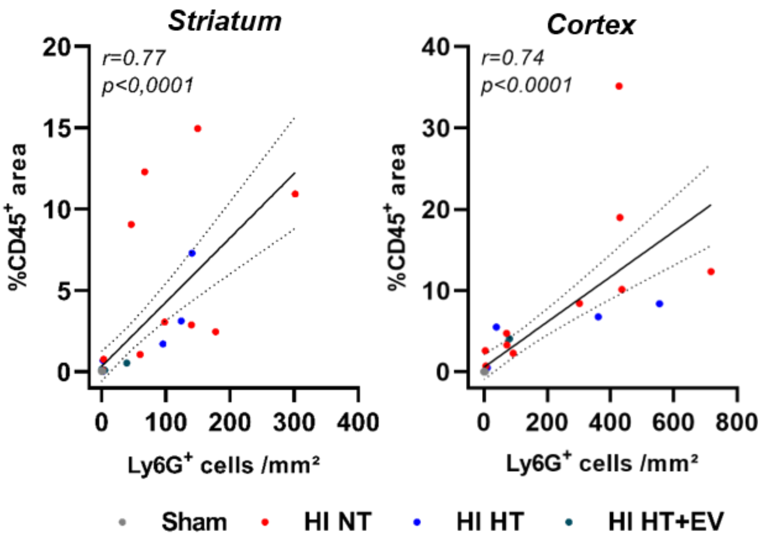

Figure S3: Correlation between neutrophil and total leukocyte accumulation. C57BL/6 mice were exposed to HI on postnatal day 9 (P9) followed by 4 h HT or NT. ciMSC-EVs were delivered intra-nasally on day 1, 3 and 5 after HI. Leukocyte accumulation and neutrophil infiltration was de-termined in CD45 and Ly6G-stained tissue sections 7 days after HI. Due to intense accu-mulation of total leukocytes in severely affected animals, the CD45 positively stained area was measured, while distinct neutrophils could be discri-minated and were counted for quantification. Correlation analyses between both outcome measures were performed for the striatum and cortex. r= Spearmann's rank correlation coefficient

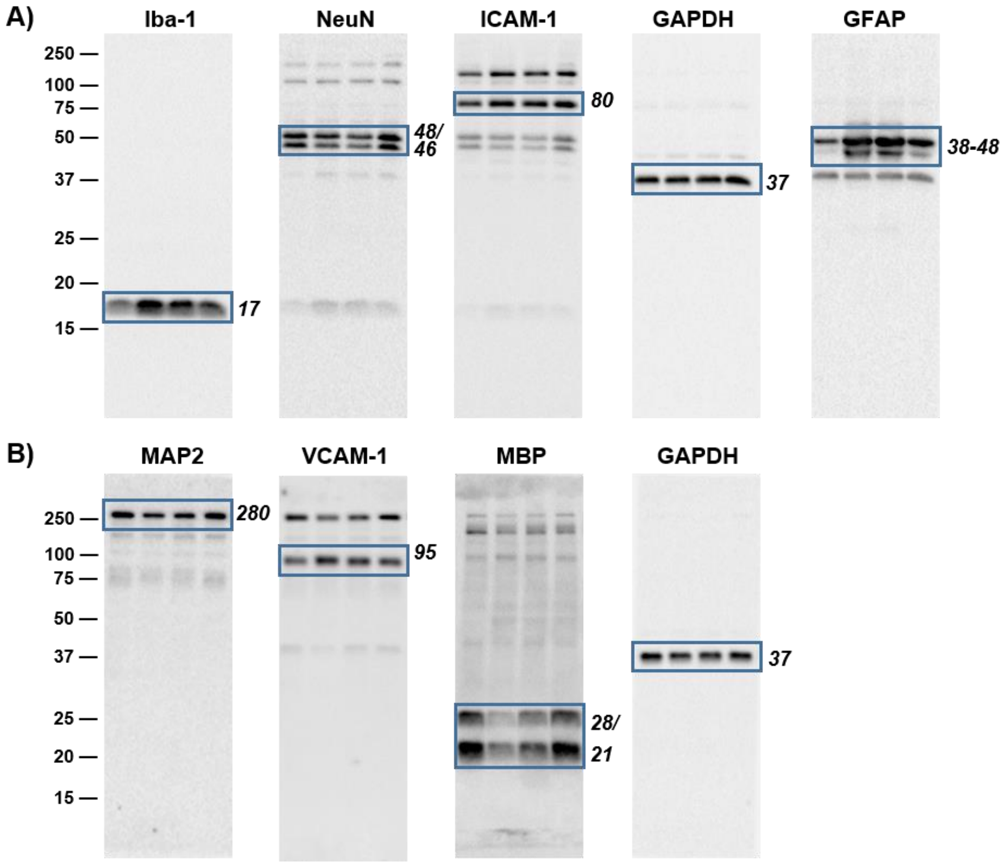

Figure S4: Original full-length western blot images used for representative illustrations. Analyses of Iba-1, NeuN, ICAM-1, GAPDH and GFAP were performed on the same membrane **(A)**. Analyses of MAP2, VCAM-1, MBP and GAPDH were performed on a same membrane **(B)**. Antibody incubation (without stripping) and detection were performed in the depicted order. Proteins were separated via electrophoreses on either 12.5% **(A)** or gradient **(B)** SDS gels. Blue boxes indicate bands used for illustration and quantification at the indicated molecular weights, based on published molecular weights for the applied antibodies (Table S4).

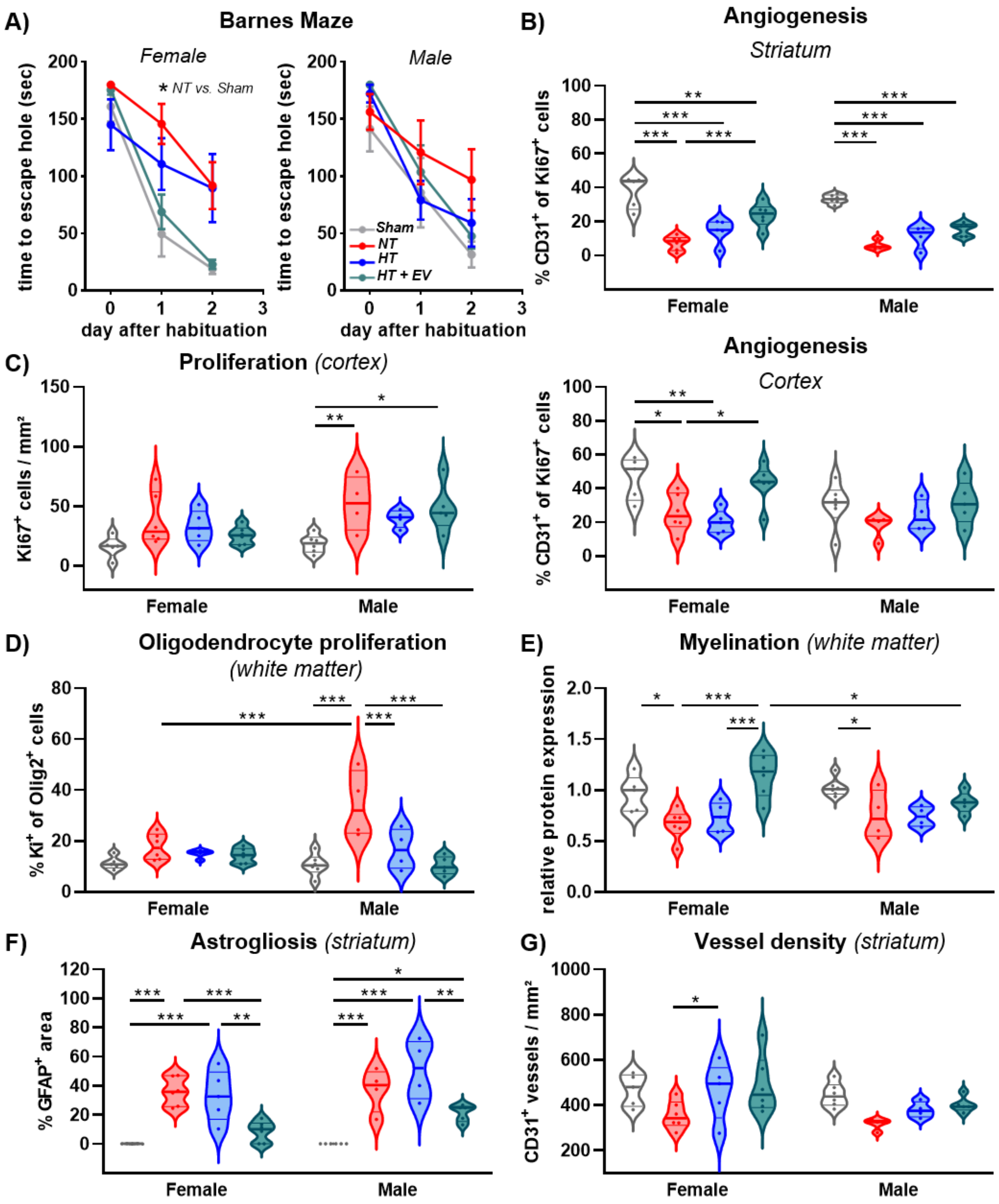

Figure S5: Graphical presentation of results from sex-stratified analyses for outcome measures with main *sex* and *treatment x sex* interaction effects. For all outcome measures, sex-stratified analyses were performed with two-way ANOVA analyses (Tables S5, 6). Sex-stratified data presentation is shown for outcomes with significant results for main sex and interaction effects between treatment and sex (highlighted in red in Tables S5, 6). Data in **A)** derived from assessment of long-term behavioural outcome 5 weeks after HI are presented as mean ± SEM (n = 6 (each sex, sham), n = 6 (f, NT), n = 7 (m, NT), n = 6 (f, HT), n = 7 (m, HT), n = 7 (each sex, HT+EV)). Data in **B) – G)** derived from immunohistochemistry analyses (B, C, D, F, G) and western blot analyses (E) 7 days after HI are presented as violin blots with individual data points (n = 5 (f, sham), n = 6 (m, sham), n = 6 (f, NT), n = 4 (m, NT), n = 5 (f, HT), n = 4 (m, HT), n = 6 (f, HT+EV), n = 5 (m, HT+EV)). *p<0.05, **p<0.01, ***p<0.001 (results of post hoc analyses correcting for multiple comparisons via Sidak's test). NT = HI + normothermia/vehicle, HT = HI + hypothermia/vehicle, HT+EV = HI + hypothermia/ciMSC-EV.

Supplementary Tables

Table S1: Group allocation and mortality

Readout/ experimental groups	female	male	mortality
Immunohistochemistry/real time PCR/western blot 7 days after HI (6 litters)			
Sham	5	6	
HI / NT / vehicle	7	5	1f, 1m *
HI / HT / vehicle	6	5	1f, 1m *
HI / HT / ciMSC-EV	6	7	2m *
Behavioral outcome 5 weeks after HI (8 litters)			
Sham	6	6	
HI / NT / vehicle	8	8	2f, 1m[#]
HI / HT / vehicle	6	8	1m*
HI / HT / ciMSC-EV	7	8	1m*

** animals died during hypoxia, i.e. after randomization and before interventions*
[#] animals died during hypoxia, except of 1 female that died after weaning

Table S2: Antibodies used for immunohistochemistry

Antigen	dilution	reactivity	host	Supplier	catalog number
NeuN	1:500	mouse	rabbit	Millipore	ABN78
CD31	1:100	mouse	rat	BD Biosciences	550274
Laminin	1:250	mouse	rabbit	Novus Biologicals	NB300-144
Olig2	1:100	mouse	rabbit	Millipore	AB9610
Olig2*	1:100	mouse	mouse	Millipore	MABN50
Ki67	1:250	mouse/rat	rabbit	Abcam	ab66155
Ki67**	1:100	mouse	rat	Thermo Scientific	14-5698-82
APC-CC1	1:100	mouse/rat	mouse	Calbiochem	OP80
PDGFR-alpha	1:100	mouse/rat	rabbit	CellSignaling	3164S
Iba-1	1:500	mouse/rat	rabbit	Wako	019-19741
GFAP	1:500	mouse	rat	Invitrogen	13-0300
CD45	1:100	mouse	rat	BD Pharmingen	550539
Ly6G	1:100	mouse	Rat	BD Pharmingen	551459

** for co-labelling with anti-PDGFR-alpha*
***for co-labelling with anti-Olig2 (mouse)*

Table S3: TaqMan Assays used for mRNA expression analyses

Gene	Assay ID
Bdnf	Mm01334043_m1
beta-2 microglobulin	Mm00437762_m1
cc1	Mm00545877_m1
Cnpase	Mm01306640_m1
Egf	Mm00438696_m1
il-1 beta	Mm00434228_m1
il-4	Mm00445259_m1
Mbp	Mm01266402_m1
tgf beta	Mm01178820_m1
Vegf	Mm00437306_m1

Table S4: Antibodies used for western blot analyses

Antigen	dilution	reactivity	host	supplier	catalog number
MAP2	1:1000	mouse	mouse	Sigma	M9942
VCAM	1:1000	mouse	goat	R&D	AF643
MBP	1:3000	mouse	mouse IgG2b	Covance	SMI 99
Iba-1	1:1000	mouse/rat	rabbit	Wako	016-20001
NeuN	1:2000	mouse/rat	rabbit	Millipore	ABN78
ICAM	1:10000	mouse	goat	R&D	BAF796
GFAP	1:5000	mouse	mouse IgG2b	Covance	SMI 22
GAPDH	1:2000	mouse	Mouse IgM	Sigma	G8795

Table S5: Two-way ANOVA results for sex-stratified analyses of data from main Figures 1 - 3

Fig. 1: Elevated Plus Maze

	velocity		distance		time in open arms		% distance in open arms		head dipping	
**interaction**	F (3. 44) = 0.9752	P=0.4131	F (3. 44) = 0.8458	P=0.4763	F (3. 44) = 0.5399	P=0.6575	F (3. 44) = 0.3725	P=0.7733	F (3. 44) = 1.074	P=0.3699
**sex**	F (1. 44) = 0.06167	P=0.8050	F (1. 44) = 0.007466	P=0.9315	F (1. 44) = 1.255	P=0.2686	F (1. 44) = 3.261	P=0.0778	F (1. 44) = 0.1380	P=0.7121
**treatment**	*F (3. 44) = 4.449*	*P=0.0082*	*F (3. 44) = 4.946*	*P=0.0048*	*F (3. 44) = 8.638*	*P=0.0001*	*F (3. 44) = 4.867*	*P=0.0052*	*F (3. 44) = 4.238*	*P=0.0102*

Fig. 1: Barnes Maze (learning. 3-way repeated measurement ANOVA)

	sham vs. NT		sham vs. HT		sham vs. HT + EV		NT vs. HT		NT vs. HT + EV		HT vs. HT+ EV	
**time**	*F (1.807. 37.95) = 34.22*	*P<0.0001*	*F (1.929. 40.52) = 46.62*	*P<0.0001*	*F (1.490. 32.77) = 89*	*P<0.0001*	*F (1.617. 35.58) = 24.39*	*P<0.0001*	*F (1.699. 39.08) =*	*P<0.0001*	*F (1.856. 42.68) = 74.22*	*P<0.0001*
**sex**	**F (1. 21) = 12.74**	**P=0.0018**	F (1. 21) = 3.625	P=0.0707	F (1. 22) = 3.585	P=0.0715	F (1. 22) = 1.977	P=0.1737	**F (1. 23) = 7.221**	**P=0.0132**	F (1. 23) = 0.5830	P=0.4529
**treatment**	F (1. 21) = 0.02897	P=0.8665	F (1. 21) = 0.003567	P=0.9529	F (1. 22) = 2.552	P=0.1244	F (1. 22) = 0.6535	P=0.4275	F (1. 23) = 0.08153	P=0.7778	F (1. 23) = 0.1576	P=0.6950
**time x sex**	F (2. 42) = 2.897	P=0.0663	F (2. 42) = 1.754	P=0.1855	F (2. 44) = 0.3292	P=0.7212	F (2. 44) = 0.8222	P=0.4461	**F (2. 46) = 6.149**	**P=0.0043**	**F (2. 46) = 4.706**	**P=0.0138**
**time x treatment**	F (2. 42) = 0.9610	P=0.3908	F (2. 42) = 0.1901	P=0.8276	F (2. 44) = 2.276	P=0.1146	F (2. 44) = 0.8653	P=0.4279	F (2. 46) = 0.6944	P=0.5045	F (2. 46) = 0.5332	P=0.5903
**sex x treatment**	F (1. 21) = 0.7329	P=0.4016	F (1. 21) = 0.5211	P=0.4783	F (1. 22) = 0.3586	P=0.5554	F (1. 22) = 0.009103	P=0.9249	F (1. 23) = 2.254	P=0.1469	F (1. 23) = 1.735	P=0.2008
**time x sex x treatmen**	F (2. 42) = 0.8595	P=0.4307	**F (2. 42) = 3.662**	**P=0.0342**	F (2. 44) = 0.1973	P=0.8217	F (2. 44) = 1.866	P=0.1668	F (2. 46) = 0.5040	P=0.6074	**F (2. 46) = 3.266**	**P=0.0472**

Fig. 1: Barnes Maze (probe trial)

**interaction**	F (3. 44) = 0.7307	P=0.5392
**sex**	F (1. 44) = 0.1614	P=0.6898
**treatment**	*F (3. 44) = 8.858*	*P=0.0001*

Suppl. Fig. 2: Open Field

	velocity		distance		time in centre	
**interaction**	F (3. 44) = 0.1354	P=0.9384	F (3. 44) = 0.1498	P=0.9293	F (3. 44) = 0.1346	P=0.9389
**sex**	F (1. 44) = 0.1668	P=0.6849	F (1. 44) = 0.1217	P=0.7289	F (1. 44) = 0.03595	P=0.8505
**treatment**	*F (3. 44) = 4.118*	*P=0.0117*	*F (3. 44) = 3.877*	*P=0.0152*	F (3. 44) = 2.049	P=0.1208

Fig. 2

	NeuN (Western Blot)		NeuN (IHC. striatum)		NeuN (IHC. cortex)		MAP2 (Western Blot)	
**interaction**	F (3. 33) = 1.035	P=0.3897	F (3. 33) = 0.1195	P=0.9480	F (3. 33) = 1.440	P=0.2489	F (3. 33) = 1.629	P=0.2015
**sex**	F (1. 33) = 0.3219	P=0.5743	F (1. 33) = 0.5542	P=0.4619	F (1. 33) = 1.593	P=0.2157	F (1. 33) = 1.241	P=0.2733
**treatment**	*F (3. 33) = 12.50*	*P<0.0001*	*F (3. 33) = 8.807*	*P=0.0002*	*F (3. 33) = 3.298*	*P=0.0324*	*F (3. 33) = 5.275*	*P=0.0044*

Fig. 3

	CD31 (IHC striatum)		CD31 (IHC cortex)		Laminin (IHC striatum)		Laminin (IHC cortex)	
**interaction**	F (3. 33) = 0.4166	P=0.7422	F (3. 33) = 0.4435	P=0.7235	F (3. 33) = 0.2398	P=0.8680	F (3. 33) = 0.2567	P=0.8560
**sex**	**F (1. 33) = 5.246**	**P=0.0285**	F (1. 33) = 3.008	P=0.0922	F (1. 33) = 1.154	P=0.2905	F (1. 33) = 2.357	P=0.1342
**treatment**	*F (3. 33) = 4.671*	*P=0.0079*	*F (3. 33) = 4.218*	*P=0.0125*	*F (3. 33) = 9.022*	*P=0.0002*	*F (3. 33) = 10.83*	*P<0.0001*
	**Ki67 (IHC striatum)**		**Ki67 (IHC cortex)**		**% CD31 of Ki67 (IHC striatum)**		**% CD31 of Ki67 (IHC cortex)**	
**interaction**	F (3. 33) = 1.753	P=0.1754	F (3. 33) = 0.9531	P=0.4263	F (3. 33) = 0.6773	P=0.57	F (3. 33) = 1.363	P=0.27
**sex**	F (1. 33) = 1.459	P=0.2357	**F (1. 33) = 5.932**	**P=0.0204**	**F (1. 33) = 5.351**	**P=0.03**	**F (1. 33) = 5.073**	**P=0.03**
**treatment**	*F (3. 33) = 5.328*	*P=0.0042*	*F (3. 33) = 6.768*	*P=0.0011*	*F (3. 33) = 45.19*	*P<0.001*	*F (3. 33) = 6.916*	*P<0.001*

IHC: immunohistochemistry

blue: significant main effects for factor treatment

red: significant main effects for sex and interaction effects between sex and treatment, these data were used for graphical presentation in Fig. S5

Table S6: Two-way ANOVA results for sex-stratified analyses of data from main Figures 4 - 6

Fig. 4

	ICAM-1 (Western Blot)		VCAM-1 (Western Blot)		CD45 (IHC striatum)		CD45 (IHC cortex)		Ly6G (IHC striatum)		Ly6G (IHC cortex)	
**interaction**	F (3. 33) = 1.956	P=0.14	F (3. 33) = 0.05771	P=0.98	F (3. 33) = 2.440	P=0.08	F (3. 33) = 0.7445	P=0.53	F (3. 33) = 0.5753	P=0.64	F (3. 33) = 1.113	P=0.36
**sex**	F (1. 33) = 0.03418	P=0.85	F (1. 33) = 2.207	P=0.15	F (1. 33) = 2.229	P=0.14	F (1. 33) = 2.155	P=0.15	F (1. 33) = 1.857	P=0.18	F (1. 33) = 3.361	P=0.08
**treatment**	*F (3. 33) = 18.03*	*P<0.001*	*F (3. 33) = 6.200*	*P=0.002*	*F (3. 33) = 11.58*	*P<0.001*	*F (3. 33) = 8.011*	*P<0.001*	*F (3. 33) = 8.923*	*P<0.001*	*F (3. 33) = 6.988*	*P<0.001*

Fig. 5

	Iba-1 (Western Blot		Iba-1 (IHC striatum)		Iba-1 (IHC cortex)		GFAP (Western Blot)	
**interaction**	F (3. 33) = 0.4981	P=0.69	F (3. 33) = 0.4463	P=0.72	F (3. 33) = 1.223	P=0.32	F (3. 33) = 2.088	P=0.12
**sex**	F (1. 33) = 0.01734	P=0.90	F (1. 33) = 0.8757	P=0.36	F (1. 33) = 0.9598	P=0.33	F (1. 33) = 0.01830	P=0.89
**treatment**	*F (3. 33) = 16.61*	*P<0.001*	*F (3. 33) = 5.266*	*P=0.004*	*F (3. 33) = 5.102*	*P=0.005*	*F (3. 33) = 16.81*	*P<0.001*
	**GFAP (IHC striatum)**		**GFAP (IHC cortex)**		**IL-1 beta**		**IL-4**	
**interaction**	F (3. 33) = 1.582	P=0.21	F (3. 33) = 1.447	P=0.25	F (3. 32) = 1.900	P=0.15	F (3. 32) = 0.4623	P=0.71
**sex**	**F (1. 33) = 5.375**	**P=0.03**	F (1. 33) = 1.990	P=0.17	F (1. 32) = 1.399	P=0.25	F (1. 32) = 0.5971	P=0.45
**treatment**	*F (3. 33) = 31.25*	*P<0.001*	*F (3. 33) = 19.77*	*P<0.001*	*F (3. 32) = 6.617*	*P=0.001*	*F (3. 32) = 46.07*	*P<0.001*
	**TGF-beta**		**BDNF**		**EGF**		**VEGF**	
**interaction**	F (3. 32) = 1.997	P=0.13	F (3. 32) = 0.2669	P=0.85	F (3. 32) = 0.4900	P=0.69	F (3. 32) = 0.8465	P=0.48
**sex**	F (1. 32) = 2.266	P=0.14	F (1. 32) = 0.6900	P=0.41	F (1. 32) = 2.145	P=0.15	F (1. 32) = 2.413	P=0.13
**treatment**	*F (3. 32) = 24.74*	*P<0.001*	*F (3. 32) = 16.95*	*P<0.001*	*F (3. 32) = 22.20*	*P<0.001*	*F (3. 32) = 57.66*	*P<0.001*

Fig. 6

	Olig2		% Ki67 of Olig2		% PDGFR of Olig2		% CC1 of Olig2	
**interaction**	F (3. 33) = 1.533	P=0.22	**F (3. 33) = 6.395**	**P=0.002**	F (3. 33) = 1.572	P=0.21	F (3. 33) = 0.8544	P=0.47
**sex**	F (1. 33) = 0.007542	P=0.93	F (1. 33) = 3.507	P=0.07	F (1. 33) = 0.06492	P=0.80	F (1. 33) = 0.9080	P=0.35
**treatment**	*F (3. 33) = 5.086*	*P=0.005*	*F (3. 33) = 14.30*	*P<0.001*	*F (3. 33) = 13.21*	*P<0.001*	*F (3. 33) = 11.11*	*P<0.001*
	**CC1 (PCR)**		**CNPase (PCR)**		**MBP (PCR)**		**MBP (Western Blot)**	
**interaction**	F (3. 32) = 1.480	P=0.24	F (3. 32) = 0.3860	P=0.76	F (3. 32) = 0.6816	P=0.57	**F (3. 33) = 2.906**	**P=0.05**
**sex**	F (1. 32) = 3.252	P=0.08	F (1. 32) = 1.390	P=0.25	F (1. 32) = 2.532	P=0.12	F (1. 33) = 0.3235	P=0.57
**treatment**	*F (3. 32) = 56.35*	*P<0.001*	*F (3. 32) = 24.66*	*P<0.001*	*F (3. 32) = 23.05*	*P<0.001*	*F (3. 33) = 10.77*	*P<0.001*

IHC: immunohistochemistry

blue: significant main effects for factor treatment

red: significant main effects for sex and interaction effects between sex and treatment, data were used for graphical presentation in Fig. S5

Table S7: Median, IQR values and results from post-hoc multiple comparisons tests of sex-stratified analyses of data from main Figures 1 - 3

Fig. 1: Elevated Pluze Maze	velocity				distance				time in open arms				% distance in open arms				head dipping			
	Female		Male		Female		Male		Female		Male		Female		Male		Female		Male	
	Median	IQR	Median	IQR	Median	IQR	Median	IQR	Median	IQR	Median	IQR	Median	IQR	Median	IQR	Median	IQR	Median	IQR
Sham	3.380	0.993	3.134	1.442	1014.00	298.10	877.40	124.50	139.60	75.30	138.70	36.90	49.86	16.50	46.96	7.11	0.2361	0.0756	0.2349	0.0189
NT	4.242	2.103	2.927	0.415	1273.00	631.50	1224.00	624.90	202.80	62.00	198.70	83.20	59.30	18.09	52.76	21.24	0.2812	0.0617	0.2769	0.0985
HT	3.931	1.754	4.080	2.084	1179.00	409.00	1335.00	254.00	201.70	57.90	188.80	63.70	61.12	18.66	54.54	16.74	0.2954	0.0603	0.2904	0.0802
HT + EV	3.134	1.442	4.451	0.846	940.10	480.60	1087.00	265.30	160.80	77.93	159.20	30.40	50.13	6.55	47.92	12.53	0.2261	0.0917	0.2757	0.0686
sign. diff. post hoc analyses			* Sham vs. HT				* Sham vs. HT		* Sham vs. NT & HT<br>* HT+EV vs. NT & HT		* Sham vs. HT								* Sham vs. HT	

Fig. 1: Barnes Maze	d0				d1				d2				probe trial			
	Female		Male		Female		Male		Female		Male		Female		Male	
	Median	IQR	Median	IQR	Median	IQR	Median	IQR	Median	IQR	Median	IQR	Median	IQR	Median	IQR
Sham	180.00	46.50	156.00	80.00	31.50	57.50	52.00	150.00	16.00	19.75	20.50	44.00	12.00	6.00	18.00	28.75
NT	180.00	0.00	180.00	68.00	164.50	84.25	156.00	161.00	74.50	77.55	95.00	153.00	47.00	61.25	47.00	40.00
HT	180.00	105.25	180.00	0.00	87.00	108.50	69.00	64.00	63.00	150.75	45.00	30.00	31.50	23.75	26.00	27.00
HT + EV	180.00	3.00	180.00	0.00	49.50	64.00	93.00	144.00	21.00	19.75	40.00	51.00	14.00	8.00	12.00	23.00
sign. diff. post hoc analyses					* Sham vs. NT								* Sham vs. NT<br>* NT vs. HT+EV		* NT vs. HT+EV	

Suppl. Fig. 2: Open Field	velocity				distance				time in centre			
	Female		Male		Female		Male		Female		Male	
	Median	IQR	Median	IQR	Median	IQR	Median	IQR	Median	IQR	Median	IQR
Sham	5.33	1.35	5.21	2.39	1599.00	401.00	1563.00	718.00	3.37	3.62	4.77	4.32
NT	6.29	2.37	5.67	3.30	1886.00	710.00	1700.00	963.00	3.27	17.80	6.40	14.26
HT	6.40	4.48	6.23	3.14	1921.00	1366.00	1869.00	942.00	5.63	5.38	3.53	7.73
HT + EV	5.76	1.97	5.12	1.42	1729.00	592.00	1537.00	425.00	3.67	3.13	1.67	3.07
sign. diff. post hoc												

Fig. 2: Western Blot & IHC	NeuN (WB)				MAP2 (WB)				NeuN (striatum)				NeuN (cortex)			
	Female		Male		Female		Male		Female		Male		Female		Male	
	Median	IQR	Median	IQR	Median	IQR	Median	IQR	Median	IQR	Median	IQR	Median	IQR	Median	IQR
Sham	0.979	0.129	1.029	0.064	0.931	0.422	1.000	0.498	1545.00	250.00	1522.00	397.00	1190.00	359.00	1235.00	173.00
NT	0.714	0.154	0.810	0.180	0.603	0.820	0.605	0.406	1091.00	503.70	970.30	616.70	1215.00	298.00	1267.00	587.40
HT	0.816	0.284	0.586	0.437	1.018	0.444	0.818	0.112	1176.00	446.80	1079.00	888.20	1470.00	171.00	1136.00	466.60
HT + EV	0.947	0.276	0.877	0.181	1.281	0.494	0.964	0.241	1372.00	297.00	1380.00	271.00	1460.00	385.00	1479.00	494.00
sign. diff. post hoc analyses	* Sham vs. NT & HT<br>* HT+EV vs. NT & HT		*** Sham vs. HT		** NT vs. HT+EV				* Sham vs. NT		* Sham vs. NT & HT					

Fig. 3: IHC	CD31 (striatum)				CD31 (cortex)				Laminin (striatum)				Laminin (cortex)			
	Female		Male		Female		Male		Female		Male		Female		Male	
	Median	IQR	Median	IQR	Median	IQR	Median	IQR	Median	IQR	Median	IQR	Median	IQR	Median	IQR
Sham	480.00	139.40	437.90	91.10	521.00	102.10	524.30	71.80	134.60	59.60	154.10	40.40	140.40	44.00	146.40	15.20
NT	341.40	102.30	325.40	43.20	427.30	108.60	401.90	114.00	286.90	122.70	314.60	84.10	305.00	133.00	285.90	328.90
HT	495.40	222.40	374.90	67.20	530.40	232.20	455.20	101.90	136.40	186.60	205.00	215.80	148.70	120.00	228.80	114.60
HT + EV	445.30	208.30	393.10	52.00	510.80	156.00	448.60	170.20	148.10	49.20	174.50	87.80	151.90	47.10	184.30	104.60
sign. diff. post hoc analyses	* NT vs. HT								** Sham vs. NT<br>** NT vs. HT+EV		* Sham vs. NT		* Sham vs. NT<br>* NT vs. HT & HT+EV		** Sham vs. NT<br>* NT vs. HT+EV	

Fig. 3: IHC	Ki67 (striatum)				Ki67 (cortex)				% CD31 of Ki67 (striatum)				% CD31 of Ki67 (cortex)			
	Female		Male		Female		Male		Female		Male		Female		Male	
	Median	IQR	Median	IQR	Median	IQR	Median	IQR	Median	IQR	Median	IQR	Median	IQR	Median	IQR
Sham	31.44	25.95	32.90	16.04	16.56	13.40	18.96	12.45	43.61	17.35	33.22	4.41	51.51	23.96	31.77	16.15
NT	41.20	70.00	111.80	110.07	28.67	39.64	52.45	44.28	8.46	7.64	4.99	4.98	23.52	19.95	20.96	10.89
HT	96.72	62.66	51.90	55.97	31.59	24.46	40.86	13.81	15.07	11.18	13.47	12.06	19.87	12.34	21.41	17.05
HT + EV	57.74	15.83	87.11	36.26	25.61	13.81	44.30	31.29	24.63	10.06	17.14	7.35	43.88	12.33	30.66	22.69
sign. diff. post hoc analyses			* Sham vs. NT				* Sham vs. NT and HT+EV		*** Sham vs. NT & HT<br>** Sham vs. HT+EV<br>*** NT vs. HT+EV		*** Sham vs. NT & HT & HT+EV		* Sham vs. NT<br>** Sham vs. HT<br>* HT vs. HT+EV			

NT: HI + normothermia/vehicle
HT: HI + hypothermia/vehicle
HT + EV: HI + hypothermia/ciMSC-EVs
IQR: interquartile range
IHC: immunohistochemistry

* p<0.05, ** p<0.01, *** p<0.001

Table S8: Median, IQR values and results from post-hoc multiple comparisons tests of sex-stratified analyses of data from main Figures 4 - 6

Fig. 4: Western Blot & IHC	ICAM-1 (WB)				VCAM-1 (WB)				CD45 (striatum)			
	Female		Male		Female		Male		Female		Male	
	Median	IQR	Median	IQR	Median	IQR	Median	IQR	Median	IQR	Median	IQR
Sham	1.000	0.141	1.033	0.146	1.003	0.204	1.086	0.266	0.022	0.035	0.016	0.031
NT	1.326	0.379	1.301	0.318	1.351	0.652	1.587	0.133	2.665	4.766	9.986	10.898
HT	1.239	0.294	1.162	0.107	1.094	0.651	1.372	0.578	0.013	3.991	0.864	2.757
HT + EV	0.943	0.529	1.010	0.065	1.151	0.875	1.103	0.399	0.007	0.006	0.094	0.326
sign. diff. post hoc analyses	*** Sham vs. NT<br>* Sham vs. HT<br>*** HT+EV vs. NT & HT		* Sham vs. NT<br>* NT vs. HT+EV		* Sham vs. NT						*** Sham vs. NT<br>*** NT vs. HT+EV<br>** HT vs. HT+EV	

Fig. 4: Western Blot & IHC	CD45 (cortex)				Ly6G (striatum)				Ly6G (cortex)			
	Female		Male		Female		Male		Female		Male	
	Median	IQR	Median	IQR	Median	IQR	Median	IQR	Median	IQR	Median	IQR
Sham	0.016	0.023	0.021	0.034	0.000	1.387	0.000	0.231	0.000	1.974	0.000	0.000
NT	5.522	10.463	8.531	25.770	82.640	146.959	105.000	214.390	196.500	425.976	249.100	573.880
HT	0.002	2.999	3.384	7.965	1.851	70.806	48.690	115.843	0.790	24.040	181.100	506.403
HT + EV	0.018	0.022	0.083	2.094	0.000	0.928	0.928	21.297	0.000	1.381	1.579	40.661
sign. diff. post hoc analyses			** Sham vs. NT<br>** NT vs. HT+EV				** Sham vs. NT<br>** NT vs. HT+EV				* Sham vs. NT<br>* NT vs. HT+EV	

Fig. 5: Western Blot & IHC	Iba-1 (WB)				GFAP (WB)				Iba-1 (striatum)			
	Female		Male		Female		Male		Female		Male	
	Median	IQR	Median	IQR	Median	IQR	Median	IQR	Median	IQR	Median	IQR
Sham	1.000	0.097	1.051	0.166	1.000	0.220	1.190	0.411	0.176	0.299	0.117	0.167
NT	2.563	1.650	2.257	0.796	5.481	4.311	4.496	2.187	6.562	19.660	9.138	15.102
HT	1.820	0.934	1.730	0.559	4.913	2.951	4.004	2.573	4.939	22.107	20.300	37.361
HT + EV	1.044	0.418	1.318	0.832	2.173	2.585	3.094	3.622	0.713	1.946	4.709	7.166
sign. diff. post hoc analyses	*** Sham vs. NT<br>* Sham vs. HT<br>*** NT vs. HT+EV		** Sham vs. NT<br>* NT vs. HT+EV		*** Sham vs. NT<br>** Sham vs. HT<br>*** NT vs. HT+EV<br>* HT vs. HT+EV		** Sham vs. NT<br>* Sham vs. HT & HT+EV				* Sham vs. NT	

Fig. 5: Western Blot & IHC	Iba- 1 (cortex)				GFAP (striatum)				GFAP (cortex)			
	Female		Male		Female		Male		Female		Male	
	Median	IQR	Median	IQR	Median	IQR	Median	IQR	Median	IQR	Median	IQR
Sham	0.353	0.650	0.255	0.333	0.130	0.209	0.120	0.070	0.098	0.121	0.099	0.098
NT	12.670	23.110	7.752	6.686	35.830	21.360	40.380	27.540	31.450	18.300	23.580	30.450
HT	1.330	7.215	3.260	6.021	32.510	32.500	52.120	39.360	12.920	16.954	31.300	35.895
HT + EV	0.258	0.601	0.931	2.631	10.050	14.342	24.800	10.400	2.075	4.651	7.873	9.249
sign. diff. post hoc analyses	** Sham vs. NT<br>* NT vs. HT<br>** NT vs. HT+EV				*** Sham vs. NT & HT<br>*** NT vs. HT+EV<br>** HT vs. HT+EV		*** Sham vs. NT & HT<br>* Sham vs. HT+EV<br>** HT vs. HT+EV		*** Sham vs. NT<br>* NT vs. HT<br>*** NT vs. HT+EV		*** Sham vs. NT & HT<br>* NT vs. HT+EV<br>* HT vs. HT+EV	

Fig. 5: mRNA expression	IL-1 beta				IL-4				TGF-beta			
	Female		Male		Female		Male		Female		Male	
	Median	IQR	Median	IQR	Median	IQR	Median	IQR	Median	IQR	Median	IQR
Sham	1.000	0.251	1.000	0.460	1.000	0.012	1.000	0.110	1.000	0.215	1.000	0.063
NT	20.300	29.420	16.300	23.754	0.050	0.079	0.132	0.103	0.625	0.230	0.449	0.174
HT	2.221	5.161	16.560	48.363	0.147	0.283	0.307	0.267	0.482	0.072	0.624	0.276
HT + EV	1.264	0.322	1.395	2.781	0.257	0.433	0.489	0.360	0.802	0.449	0.632	0.230
sign. diff. post hoc analyses	* Sham vs. NT<br>* NT vs. HT+EV		* Sham vs. HT		*** Sham vs. NT & HT & HT+EV<br>* NT vs. HT+EV		*** Sham vs. NT & HT<br>* NT vs. HT+EV		*** Sham vs. NT & HT<br>** HT vs. HT+EV		*** Sham vs. NT & HT & HT+EV	

Fig. 5: mRNA expression	BDNF				EGF				VEGF			
	Female		Male		Female		Male		Female		Male	
	Median	IQR	Median	IQR	Median	IQR	Median	IQR	Median	IQR	Median	IQR
Sham	1.000	0.044	1.000	0.243	1.000	0.218	1.000	0.159	1.000	0.043	1.000	0.118
NT	0.180	0.618	0.174	0.175	0.288	0.399	0.180	0.389	0.141	0.142	0.140	0.053
HT	0.406	0.244	0.209	0.461	0.363	0.205	0.236	0.247	0.351	0.223	0.180	0.351
HT + EV	0.740	0.657	0.830	0.491	0.869	0.473	0.648	0.473	0.584	0.462	0.467	0.387
sign. diff. post hoc analyses	** Sham vs. NT & HT		*** Sham vs. NT<br>** Sham vs. HT<br>* NT vs. HT+EV		*** Sham vs. NT & HT<br>** HT+EV va. NT & HT		*** Sham vs. NT & HT<br>* Sham vs. HT+EV		** Sham vs. NT & HT & HT-EV<br>*** NT vs. HT+EV<br>* HT vs. HT+EV		*** Sham vs. NT & HT & HT+EV	

Fig. 6: IHC (white matter)	Olig2				% Ki67 of Olig2				% PDGFR of Olig2				% CC1 of Olig2			
	Female		Male		Female		Male		Female		Male		Female		Male	
	Median	IQR	Median	IQR	Median	IQR	Median	IQR	Median	IQR	Median	IQR	Median	IQR	Median	IQR
Sham	661.60	168.20	738.80	136.50	10.77	4.25	10.38	5.89	15.90	3.37	10.13	5.40	37.49	7.13	45.36	6.63
NT	935.50	306.40	926.50	308.40	17.21	9.91	32.03	24.60	19.66	4.54	20.04	11.63	25.22	10.72	27.10	14.64
HT	833.80	96.70	705.50	95.50	15.44	2.32	16.37	15.23	17.65	6.46	19.35	9.22	31.51	5.75	30.43	26.58
HT + EV	712.90	75.80	751.80	177.00	14.45	5.93	9.64	6.67	7.31	10.38	10.33	9.00	37.75	6.22	41.65	9.79
sign. diff. post hoc analyses	** Sham vs. NT<br>** NT vs. HT+EV						*** Sham vs. NT<br>*** NT vs. HT & HT+EV		*** NT vs. HT+EV<br>* HT vs. HT+EV		* Sham vs. NT & HT<br>** HT+EV vs. NT & HT+		* Sham vs. NT<br>* NT vs. HT+EV		** Sham vs. NT & HT	

Fig. 6: mRNA expersession & Western Blot	CC1 (PCR)				CNPase (PCR)				MBP (PCR)				MBP (WB)			
	Female		Male		Female		Male		Female		Male		Female		Male	
	Median	IQR	Median	IQR	Median	IQR	Median	IQR	Median	IQR	Median	IQR	Median	IQR	Median	IQR
Sham	1.000	0.084	1.000	0.130	1.000	0.023	1.000	0.095	1.000	0.055	1.000	0.073	1.000	0.326	1.010	0.122
NT	0.133	0.054	0.111	0.029	0.228	0.552	0.161	0.064	0.213	0.527	0.144	0.082	0.688	0.186	0.718	0.449
HT	0.320	0.317	0.164	0.287	0.360	0.124	0.292	0.275	0.314	0.144	0.242	0.313	0.737	0.278	0.741	0.191
HT + EV	0.736	0.541	0.466	0.428	0.807	0.501	0.681	0.711	0.899	0.662	0.744	0.698	1.183	0.391	0.880	0.174
sign. diff. post hoc analyses	*** Sham vs. NT & HT<br>* Sham vs. HT+EV<br>*** NT vs. HT+EV<br>** HT vs. HT+EV		*** Sham vs. NT & HT & HT+EV		*** Sham vs. NT & HT<br>* Sham vs. HT+EV<br>* HT+EV vs. NT & HT		*** Sham vs. NT & HT<br>* Sham vs. HT+EV<br>* NT vs. HT+EV		*** Sham vs. NT & HT<br>** HT+EV vs. NT & HT		*** Sham vs. NT & HT<br>* Sham vs. HT+EV		* Sham vs. NT<br>*** HT+EV vs. NT & HT		* Sham vs. HT	

NT: HI + normothermia/vehicle
HT: HI + hypothermia/vehicle
HT + EV: HI + hypothermia/ciMSC-EVs
IQR: interquartile range
IHC: immunohistochemistry

* p<0.05, ** p<0.01, *** p<0.001
